# Improvement in the Surveillance System for Livestock Diseases and Antimicrobial Use Following Operational Research Studies in Sierra Leone January–March 2023

**DOI:** 10.3390/tropicalmed8080408

**Published:** 2023-08-10

**Authors:** Samuel Alie Konteh, Fatmata Isatu Bangura (Turay), Amara Leno, Srinath Satyanarayana, Divya Nair, Mohamed Alpha Bah, Salam Saidu, David Sellu-Sallu, Sahr Raymond Gborie, Sorie Mohamed Kamara, Amadu Tejan Jalloh, Joseph Sam Kanu, Kadijatu Nabie Kamara, Matilda Mattu Moiwo, Esther Dsani, Noelina Nantima

**Affiliations:** 1 Livestock and Veterinary Services Division, Ministry of Agriculture and Forestry, Youyi Building, Brooked, Freetown 00232, Sierra Leonelenoamara87@yahoo.fr (A.L.); medalphabah2014@gmail.com (M.A.B.); salamsaidu61@yahoo.com (S.S.); davidsellusallu@gmail.com (D.S.-S.); srgborie@gmail.com (S.R.G.); soriesl@yahoo.com (S.M.K.); jallohtejan770@yahoo.com (A.T.J.); 2 Center for Operational Research, International Union Against TB and Lung Disease (The Union), 75006 Paris, France; ssrinath@theunion.org (S.S.); divya.nair@theunion.org (D.N.); 3 National Disease Surveillance Program, Directorate of Health Security and Emergencies, Ministry of Health and Sanitation, Sierra Leone National Public Health Emergency Operations Centre, Cockerill, Wilkinson Road, Freetown 00232, Sierra Leone; samjokanu@yahoo.com (J.S.K.); kamarakadijatunabie@gmail.com (K.N.K.); 4 College of Medicine and Allied Health Sciences (COMAHS), University of Sierra Leone (USL), Tower Hill, Freetown 00232, Sierra Leone; 5 Joint Medical Unit, Ministry of Defense, Republic of Sierra Leone Armed Forces, Freetown 00232, Sierra Leone; 6 Emergency Center for Transboundary Animal Diseases (ECTAD), Food and Agriculture Organization of the United Nations (FAO), Freetown 00232, Sierra Leone; esther.dsani@yahoo.com (E.D.);

**Keywords:** surveillance, animal health, antimicrobial use, antimicrobial resistance, operational research, impact, dissemination, Sierra Leone, SORT IT

## Abstract

In Sierra Leone, two operational research (OR) studies in 2019 and 2021 showed deficiencies in the data being captured by the Integrated Animal Disease Surveillance and Reporting (IADSR) system. This third OR study was conducted in 2023 to assess whether the second OR study’s results and recommendations were disseminated with the key stakeholders, the uptake of the recommendations, improvements in data capture in the IADSR system, and to describe the data on livestock disease and antimicrobial use. In 2022, on seven occasions, the authors of the second OR study disseminated the study’s findings. Of the four recommendations, the one on improving laboratory infrastructure for confirmation of animal disease was not implemented. The district animal health weekly surveillance reports received through the IADSR system were sustained at 88% between the second (2021) and third (2023) studies. In both studies, the proportion of sick animals receiving antibiotics (25%) remained the same, but the use of “critically important antimicrobials for veterinary use” declined from 77% (in 2021) to 69% (in 2023). The IADSR system has improved considerably in providing information on animal health and antibiotic use, and sequential OR studies have played a key role in its improvement.

## 1. Introduction

Surveillance for diseases among livestock is essential for controlling infection and infestation, monitoring trends of livestock diseases and undertaking measures to safeguard animal and human health [1]. Inappropriate use of antimicrobials in the treatment of livestock diseases can also lead to antimicrobial resistance (AMR) that is detrimental to both animal and human health [2]. The World Organization for Animal Health (WOAH) recommends that countries should establish a harmonized national surveillance system that captures data on antimicrobial use (AMU) and AMR in relevant animal pathogens and contributes to the global monitoring database on animal health and antimicrobial agent use in livestock hosted by the WOAH [3].

In many high-resource countries, surveillance systems for monitoring AMU and AMR in livestock have been successfully established [4]. However, in spite of the presence of significant risk factors for communicable diseases and transmission of resistant pathogens, most low- and middle-income countries (LMICs) are lagging behind in AMR surveillance [4,5].

Sierra Leone, like many other LMICs, did very little to regulate the use of antimicrobial agents in livestock. A situational analysis conducted by the Government in 2017 to get baseline information on AMR found that the country had very limited information on AMR, and there was no national guidance on appropriate AMU in both humans and animals [6]. Compounding the problem was the shortage of veterinary officers and veterinary drugs, including antibiotics, with treatment of sick livestock in most parts of the country done by field livestock officers who had only basic training in veterinary medicine. The Livestock and Veterinary Services Division of the Ministry of Agriculture and Food Security has been collecting data on AMU in livestock since 2012 as part of the Epidemic-surveillance Network of Animal Diseases in Sierra Leone, which was replaced in 2019 by the Integrated Animal Disease Surveillance and Reporting (IADSR) system [7]. However, there was no formal evaluation of the process, including the quality of data collected and submitted by the field livestock officers to the Livestock and Veterinary Services Division, including the Epidemiology Unit (LVSDEU) of the Ministry of Agriculture and Food Security.

The LVSDEU, therefore, undertook an operational research study as part of the WHO’s structured operational research training initiative (SORT IT) course in 2019 (henceforth referred to as the ‘first study’) to assess the functioning of the IADSR system among livestock [8]. This first study showed several deficiencies in the completeness and timeliness of the submission of district animal health monthly surveillance reports. Only 1% of the expected 616 district animal health monthly surveillance reports were received at the national level. The results of the first study were widely disseminated with all the stakeholders, training was conducted, and the recording system was revised with the help of in-country partners such as the Food and Agriculture Organization (FAO).

1. The reporting system was changed from a monthly to a weekly system [9].
2. A follow-up second operational research study was conducted in 2021 as part of another SORT IT course to assess progress. The second study showed that 88% of the expected weekly reports were received at the national level [10]. The second study, conducted in 2022 using March to October 2021 data, provided information on animal diseases and showed that 26% of the sick animals were treated with antimicrobials, of which 77% were considered “critically important for veterinary use” by the WOAH [11]. About 17% of these antibiotics were also considered “critically important in humans” by the World Health Organization (WHO). Apart from these findings, the second study also identified further gaps in the IADSR system.

In this context, a third operational research study was undertaken in early 2023 to assess whether the results of the second study were disseminated and whether its recommendations were acted upon by the LVSDEU and the subsequent changes made in the surveillance system, and to assess whether changes have occurred in the reporting of livestock diseases and the use of antimicrobials. The specific objectives were (1) to describe the efforts made by the authors to disseminate the results of the second operational research study and the actions taken by the LVSDEU to further improve the IADSR system; and (2) to assess the changes between 2021 and 2023 in the submission of district animal health weekly surveillance reports, including the livestock diseases reported and antimicrobials used in their treatment.

## 2. Materials and Methods

### 2.1. Study Design

For objective 1, a descriptive study that involves self-reported information on the efforts made by the authors of the second study to disseminate the results to the key stakeholders of the LVSDEU and the action taken by LVSDEU for improving the IADSR system.

For objective 2, a descriptive study using secondary data published in the first and second studies and the IADSR surveillance data for the period January–March 2023.

### 2.2. Study Setting

Sierra Leone, a country in West Africa, has a population of about 8 million people [12]. Sierra Leone is bordered to the east and northwest by the Republic of Guinea and to the south by the Republic of Liberia. There are 16 administrative districts in the country, but from the Ministry of Agriculture and Food Security’s organization, there are 15 Agricultural districts. The two administrative districts, ‘western area rural’ and ‘western area urban’, of the country are considered one district headed by one District Agricultural Officer. Each district is further divided into chiefdoms, and each chiefdom into villages.

#### 2.2.1. Livestock in Sierra Leone

The Livestock and Veterinary Services Division [one of the five divisions of the Ministry of Agriculture and Food Security] is responsible for promoting animal production and animal health. The division is made up of two units: (1) the Animal Health Unit, which oversees veterinary Services, animal welfare, public health, inspection, regulations, and certification, as well as animal health and disease surveillance, including AMR; and (2) the Animal Production Unit, which oversees animal husbandry, animal traction, and animal production activities. The WOAH’s Terrestrial Animal Health Code, Chapter 6.10, which offers guidance on the responsible use of antibiotics in veterinary medicine, serves as the foundation for disease treatment in the Livestock and Veterinary Services Division.

#### 2.2.2. Animal Health Surveillance in Sierra Leone

Each agricultural district has an office of the Livestock and Veterinary Services Division. At the district level, the division has a District Livestock Officer (DLO)/District Veterinary Officer (DVO); at the chiefdom level, there are livestock assistants/inspectors (LAs/LIs), and at the community (village) level, there are community animal health workers (CAHWs). The CAHWs are expected to routinely visit farmers to examine the health of their animals and determine the number of susceptible, sick, and dead animals. The DLO, DVO, LAs/LIs and CAHWs are involved in identifying sick animals and treating them with antimicrobials when necessary. Using a standardized paper reporting format (Supplementary File S1), the CAHWs provide information regarding animal illness observed during their visits to farmers to the LAs at the chiefdom level or the DLO/DVO, depending on proximity; the LA/LI gathers the data and sends it to the DLO/DVO. Every week (Monday) before 4:00 p.m., the disease surveillance focal person compiles the data supervised by the DLO/DVO and electronically sends it to the LVSDEU using a standard epidemiological reporting format (Supplementary File S2). Data from the DLO/DVO for one week includes sub-reports from several CAHWs. The weekly livestock disease surveillance reports from the district contain data on the types of diseases, species affected, the overall number of animals at risk, method of diagnosis, animal sex, the number of sick animals, the village and chiefdom of the outbreak/disease event, the number of animals treated and/or vaccinated, the antimicrobials used in the treatment, their dosages and any other actions taken.

The district’s weekly livestock disease surveillance reports contain data on the country’s top 18 priority transboundary animal and zoonotic diseases and conditions. These are Peste des petite ruminant, rinderpest, hemorrhagic septicemia, black quarter/blackleg, contagious bovine pleuropneumonia, African swine fever, trypanosomiasis, orf (ecthyma contagiosum), brucellosis, TB, infectious caprine pleuropneumonia, Avian Influenzas, Anthrax, Newcastle disease, rabies, foot and mouth disease, and Rift Valley fever. In addition, data on six country-prioritized zoonotic diseases of national importance are also collected. These are zoonotic influenza, viral hemorrhagic fevers (Ebola and Lassa fever), salmonellosis, plague, anthrax, and rabies. At the LVSDEU, raw data from the districts are extracted, compiled, examined, and analyzed, and a PowerPoint presentation is made and shared every Wednesday with the Emergency Preparedness Response Resilience Group (EPRRG), a meeting of One Health stakeholders with participants drawn from the Ministry of Agriculture, Health and Environment and their corresponding partners. The indicators used for monitoring are like those presented in the results section in Tables 4, 7 and 8 of this manuscript. Following this activity, information is shared with various stakeholders at the national and international levels via emails and bulletins. The information is also shared with all animal health workers, including the DLO/DVO, LAs, LIs and CAHWs, and through them, the relevant information is shared with the animal farmers.

### 2.3. Study Participants/Units, Data Collection and Variables

Objective 1: The study participants included all seven representatives of the LVSDEU who took part in the second operational research study.

Objective 2: Data on the completeness and timeliness of submission of weekly livestock disease surveillance reports from the 15 districts to the national level for the year 2023 were derived from the district weekly livestock disease surveillance reports database. At each district level, for every week, we assessed the number of CAHWs/VA reports expected and received (completeness) and whether the compiled district weekly livestock disease surveillance report was submitted by the district during that week. From the district weekly livestock disease surveillance reports database, on a weekly basis, the number of animals examined, the number of sick animals identified, and the number of sick animals treated with antimicrobials by CAHWs, LAs, DLOs or DVOs during the period of January to March 2023 were determined. The antimicrobials used in the treatment of sick animals were classified as per the WOAH list of antimicrobial agents of veterinary importance [11]. Corresponding data for the year 2021 were collected from the second study [8,10].

### 2.4. Data Analysis and Interpretation

For objective 1, the data from all seven respondents were compiled and presented as a line list of activities undertaken to disseminate the results and the actions taken on the recommendations of the second study to improve the IADSR system and regulate antimicrobial use among animals. The list of observed/reported changes in the IADSR system in three domains—“data collection”, “data analysis” and “action being taken based on the data analysis”—for the years 2019, 2021 and 2023 have been described.

For objective 2, we have compiled data on the number of Animal Health Weekly Reporting forms expected and available in the year 2023 compared to the first and second studies conducted in 2019 and 2021, respectively. All quantitative data pertaining to livestock diseases and the antimicrobials used in their treatment have been summarized using frequencies and proportions.

## 3. Results

The details of the efforts made by the study investigators of the second study to disseminate the study results and its recommendations are given in Table 1. Dissemination was made through eight distinct occasions/activities to various stakeholders.

The actions taken on the key recommendations of the second operational research study for improving the IADSR system are given in Table 2. Out of the four main recommendations, three have been acted upon, and the recommendation on strengthening laboratory infrastructure for microbiological confirmation of livestock diseases has not been implemented.

The status of the IADSR system in three time periods—2019, 2021 and 2023—are listed in Table 3. Most of the improvements that were observed in 2021 in the domains of data collection, data analysis and action taken based on the data analysis were sustained in 2023.

The number of district animal health weekly reports received in 2016–2019, 2021 and 2023 are given in Table 4. Nearly 88% of the district animal health weekly reports were received in 2023, like what was observed in 2021. Of the 15 districts, three districts (Bonthe, Koinadugu and Moyamba) were not submitting the weekly reports in a timely manner.

About 10 CAHWs per district were available to report about the animal health (range 8–10) each week (Table 5). The number of CAHW reports included in the district animal health weekly reports during 2023 disaggregated by districts shows that only 49% of the 180 weekly reports contained data from all CAHWs. In eight districts (Bombali, Bonthe, Kambia, Kailahun, Kanema, Kono, Koinadugu, Moyamba), less than 50% of the reports submitted by them contained information from all the CAHWs available in their respective districts.

The status of the data quality issues in 2023 that were observed in the district animal health weekly reports in 2021 is presented in Table 6. Some inconsistencies pertaining to the numbers of susceptible versus sick versus treated and antimicrobial/anthelmintic use that were observed in 2021 district reports were not seen in 2023. However, the other inconsistencies remained, but the number of district reports showing that inconsistency declined. One major improvement has been the availability of information on the number of CAHWs in the district during the week and the number of CAHW reports that were included in the district animal health weekly reports. Availability of this information in the 2023 district animal health weekly reports enabled construction of Table 5 to assess the completeness of the surveillance data in each of the district animal health weekly reports.

The number of livestock treated with antimicrobial drugs disaggregated by the animal species in Sierra Leone during January—March 2023 compared to March—October 2021 is given in Table 7. Overall, 25% of the sick animals were treated with an antimicrobial in both 2021 and 2023. The use of antimicrobials declined among dogs, donkeys, horses and rabbits. The use of antibiotics in fowls and pigs increased.

The use of WOAH-classified “critically important antimicrobials for veterinary use” in the treatment of sick animals is given in Table 8. Overall, the proportion of critically important antimicrobials for veterinary use declined from 77% in 2021 to 69% in 2023.

## 4. Discussion

This is the first study from Sierra Leone in the animal health surveillance system to describe the changes at three different periods in the last five years. There are seven main findings. These findings have implications not only for informing policy and practice to improve the animal health surveillance system but also for understanding whether and how operational research studies support changes in the desired direction.

First, our study shows that the findings of the previous operational research study were disseminated to the various stakeholders (as envisaged), and eight distinct events could be identified. During these dissemination events, it appeared that the findings of the second operational research study were well received and decisions to act on the recommendations of the previous operational research study were made. This is a positive finding and shows that the operational research study was used to understand and address the gaps in the surveillance system. This is one of the first global studies (especially in animal health) to provide information on the dissemination efforts made by the study investigators and could become a part of the best practices for operational researchers globally.

Second, though recommendations pertaining to revising the reporting formats and provision of digital tools (such as laptops and tablets with data packages) to submit the reports were acted upon, a recommendation for improving the laboratory infrastructure for microbiological confirmation of the animal diseases under surveillance has not yet been operationalized. Obtaining microbiological confirmation is necessary to avoid misdiagnosis/misclassification of animal diseases [13]. Unlike other recommendations, acting on this recommendation requires collaboration with human health departments and additional financial resources to set up the infrastructure and coordination mechanisms [14]. Continuous advocacy with policy makers highlighting the importance of establishing the laboratory infrastructure is urgently needed to strengthen this component of the surveillance system.

Third, the improvement seen in the receipt of the expected district animal health weekly surveillance reports from 1.5% in 2019 to 88% in 2021 has been sustained in 2023. Three districts showed a decline in the submission of the weekly reports in a timely manner in 2023 when compared to 2021. Identifying and addressing the reasons for non-submission and/or delayed submission of the reports will help in improving the existing surveillance system. One big improvement in 2023 was the availability of additional information on the percentage of district weekly reports that contained data/surveillance information from all CAHWs in the respective districts. Only about 49% of the district animal health weekly surveillance reports contained surveillance data from all CAHWs. This shows that the IADSR system is still far from ideal, and there is a lot of scope for improving the completeness of the data in this system.

Fourth, a few inconsistencies that were observed in the data submitted by the district animal health weekly reports in 2021 persisted in 2023. Of particular importance is the inconsistent use of terminologies to denote livestock diseases and the antimicrobials used in their treatment, resulting in delays in compiling the reports. Addressing this inconsistency requires standardizing the terminologies for reporting, minor revisions in the reporting formats, continuous on-the-job training, supportive supervision and feedback. Use of a digital reporting platform for surveillance with structured dropdown fields can also be used to mitigate the deficiencies (instead of using paper-based and Excel-based collection and compilation) in surveillance data.

Fifth, the proportion of sick animals receiving antibiotics has remained the same, at 26%, between 2021 and 2023. There are several factors that affect the use of antibiotics in our setting. First, antibiotics are not recommended for all sicknesses; second, the owners of sick animals will have to procure and provide the antibiotics (when indicated) to their sick animals, and they may fail to do so if there are any economic constraints. Understanding which of these factors is responsible for the observed lack of change requires further analysis, which is beyond the scope of the present paper.

Sixth, the use of “critically important antimicrobials for veterinary use” shows a declining trend, from 77% in 2021 to 69% in 2023. Though this is a good sign, the use of these antibiotics is still high and needs rapid reduction [15]. Currently, there are no in-country data on whether these antibiotics were used appropriately or inappropriately. This is an area for future evaluation or research. Meanwhile, continuous engagement and advocacy with veterinary doctors are needed to optimize the prescription and consumption of these critically important antibiotics in the country.

Lastly, this study shows how operational research can be used for understanding and addressing implementation gaps and improving the quality of animal health surveillance. The optimal use of operational research under routine programmatic conditions requires human resource capacity building, and veterinary health policy makers (nationally and globally) should consider embedding a SORT IT [16] approach into their public programs for sustainable capacity building of the program personnel. This will also enable program planning and evidence-based implementation.

The major strength of the study was that it was done under routine programmatic conditions; therefore, the reported data reflect ground-level realities. The major limitations of this third study are (a) the data for the first objective were self-reported by the authors of the second operational research study, and some of them are co-authors of the present study. Therefore, there could be some reporting bias in the manner in which the findings of the second operational research study were disseminated, received by the stakeholders and/or used to make decisions; (b) despite the present study following the same methodology as that of the second operational research study for data analysis and interpretation, there were some differences in the way the same data were reported across the two study periods. Therefore, the changes observed between the 2021 and 2013 time periods are due to a combination of both actual changes and changes in the way data were reported; (c) the data on animal sickness are not laboratory confirmed; therefore, the magnitude of animal disease reported in this study needs to be interpreted with caution.

## 5. Conclusions

The present operational research study is the third sequential study to assess the status of the Integrated Animal Disease Surveillance and Reporting system in the country. Our study shows that the results and recommendations of the second operational research study of 2021 were disseminated among stakeholders, and several actions were initiated as per the study’s recommendations. Many improvements seen in the surveillance reporting during the second 2021 study were sustained in 2023. However, some of the issues observed in the reporting systems in 2021 (regarding incompleteness, inconsistency in the terms used to report the animal diseases and the antibiotics used in their treatment) persist. The recommendation to improve the laboratory infrastructure for confirmation of animal diseases (in the second study) is yet to be fully acted upon. The proportion of sick animals receiving antibiotics has remained the same. However, the use of “critically important antimicrobials for veterinary use” is showing a declining trend. Overall, the first and second operational research studies have supported significant improvement in the Integrated Animal Disease Surveillance and Reporting system. However, as shown in this third operational research study, the system is far from ideal and needs further changes to improve completeness, consistency in the terms used for reporting and laboratory confirmation of the data on animal sickness in the surveillance system.

## Figures and Tables

**Table 1:** Dissemination details of the second operational research study conducted in 2021 [10].

Mode of Delivery *	To Whom	When	Where	Decisions Made
Three-minute lightning PowerPoint presentation	Livestock staff HQ MAFS stakeholders	March 2022 April 2022 July 2022	MAFS HQ At the national SORT IT module 4 MAFS stakeholders meeting	Addition of column with new fields for gathering information on antimicrobials in the standard reporting template of animal health weekly surveillance reporting form.
Published article [10]	Global and MAFS professional groups	May 2022	Social media platforms: Whatsapp, Facebook, and LinkedIn	Disseminated with stakeholders in the livestock division
Handouts	Global and MAFS professional groups	April 2022	Social media platforms: Whatsapp, Facebook, and LinkedIn	Disseminated with stakeholders in the livestock division
Presentation (full presentation)	MAFS (DVOs/DLOs, Epi Unit, Lab technicians, district surveillance focal personnel/CAHW supervisors	8 September 2022	Port Loko	Findings were presented to all DLOs, MAFS senior technical, including the livestock division.
Presentation (full presentation)	DLOs/DVOs, Epi Unit, Lab technicians, SLARI FAO	September 2022	Port Loko	Developed Guidelines for infection prevention and appropriate antimicrobial use in the poultry sector.
Presentation (full presentation)	MAFS (DVOs/DLOs, Epi Unit, Lab technicians, SLARI)	4–6 October 2022	Port Loko	Design protocol for implementation of active surveillance for AMR in poultry to obtain measures on the status of AMR in the general population of healthy chickens to which the human population is exposed.
Presentation (lightning presentation)	MAFS (national and all regional district staff [Epi Unit, DVOs/DLOs]) MOECC/EPA (national and all regional district staff); MOHS (All district staff) /FAO/US-CDC/WHO, CDC/WHO	2 and 3 November 2022	Sierra Palms Hotel, Aberdeen, Freetown, Sierra Leone	Shared presentation among DLOs, MOHS and MOECC Cascading of findings was done by district officers to personnel.
Presentation (full presentation)	DLOs/DVOs, Epi Unit, Lab technicians, FAO	14–18 November 2022	FAO office Freetown Road Wilberforce	Profile poultry farms in selected surveillance area for antimicrobial resistance to locate poultry farms and collect AMR active surveillance data

* Dissemination material included a copy of the published article, handout, three minutes of lightning PowerPoint presentation, ten minutes of technical presentation and any other material that was taught during module 4 of the SORT IT course. These have been shared as Supplementary Files. Abbreviations: AMR—Antimicrobial Resistance; AMU—Antimicrobial Use; US-CDC—United States-Centers for Disease Control and Prevention; DLOs—District Livestock Officers; DVOs—District Veterinary Officers; Epi Unit—Epidemiology Unit; FAO—Food and Agriculture Organization; Lab technicians—Laboratory Technicians; MAFS—Ministry of Agriculture and Food Security; MOECC/EPA—Ministry of Environment and Climate Change/Environmental Protection Agency; WHO—World Health Organization.

**Table 2:** List of recommendations from the second operational research study for improving the Integrated Animal Disease Surveillance and Reporting (IADSR) system and status of actions.

Recommendation *	Action Status	Status of Implementation
Need to further improve reporting system under leadership of the Ministry of Agriculture and Food Security. Development of standard reporting formats. Inclusion of leftover monitoring indicators in reporting formats.	Addition of column with new fields for gathering information on antimicrobials in the standard reporting template of animal health weekly surveillance reporting form. Though the column has been added, there is a need for reminding the District Livestock Officers to report on these new fields.	Partially
Dedicated human resources for ensuring timely review and analysis.	Completed; five staff with qualifications in veterinary science/data science (four staff with qualification in BSc. Hons Animal Science and one staff with Doctor of Veterinary Medicine) have been recruited.	Ongoing
Capacity building of livestock officers and community workers.	Training of District Livestock Officers on animal diseases and reporting; training of laboratory technicians in animal health and human health for sample collection and microbiological methods. This training was conducted by the Senior Veterinary Officer (he is also the Head of the Epidemiology Unit, a laboratory expert from the Emergency Center for Transboundary Animal Diseases of the FAO Sierra Leone country office).	Ongoing
Strengthening laboratory infrastructure for microbiological confirmation of livestock diseases and for assessing antimicrobial resistance.	Partially completed/Partnerships developed with the human health’s Central Public Health Reference Laboratory. However, the services are yet to be operationalized.	Not implemented

* recommendations from the second operational research study [10].

**Table 3:** Status of the Integrated Animal Disease Surveillance and Reporting (IADSR) system in Sierra Leone in 2019, 2021 and 2023.

Domain	Status of IADSR System in Various Time Periods		
	2019	2021	2023
Data collection			
CAHW to DLO/DVO	Paper-based monthly, irregular reporting	Paper-based weekly	Paper-based weekly
DLO/DVO to national	Paper-based monthly, irregular reporting	Electronic regular weekly	Electronic regular weekly
Reporting formats	not structured	Structured	Structured
Mobile tablet (for data collection)	Not available	Available	Available
Internet connectivity	Not available	Available	Not available
Number of Diseases reported	16	17	17
Data analysis			
Computers for data compilation, data analysis	2 computers at national level; no computers at district level	14 desktops and 14 laptops at district level	13 desktops and 10 laptops at district level
Data on animal diseases	Data not analyzed; just submitted to OIE (WOAH)	Data analyzed and disseminated; database available, submitted to OIE (WOAH)	Data analyzed and disseminated to OIE (WOAH) and One Health platform
Data on antibiotic use	Data not analyzed; just submitted to OIE (WOAH)	Data analyzed and disseminated; database available, submitted to OIE (WOAH)	Data analyzed and disseminated; database available
Action taken based on the data analysis			
Sample collection to confirm animal disease	No Sample collection done; no confirmation done	Sample collection, analysis and confirmation done	Sample collection, analysis and confirmation done
Mapping of animal diseases for assessing distribution and clustering	Not done	Done	Done
Motorbikes and Computers	Not available	Available	Available
Cold Chain	Not available	Available	Available
Number of diseases reported	16	17	17
AMR testing at Animal Health laboratory	Not conducted	Not conducted	Not conducted

**Table 4:** The number of Animal Health Weekly Reporting forms expected and available per district in Sierra Leone in 2019, 2021 and 2023.

District	Number of Weekly Reports Received Out of Expected ^1^					
	January–December 2019 ^2^		March–October 2021 ^3^		January–March 2023	
	n	(%)	n	(%)	n	(%)
All districts	12/780	(1.5)	461/525	(88)	159/180	(88)
Bo	0/52	(0)	33/35	(97)	12/12	(100)
Bombali	1/52	(2)	35/35	(100)	12/12	(100)
Bonthe	0/52	(0)	29/35	(83)	8/12	(67)
Falaba	0/52	(0)	34/35	(97)	12/12	(100)
Kambia	3/52	(6)	31/35	(89)	12/12	(100)
Kailahun	0/52	(0)	31/35	(89)	10/12	(83)
Karene	0/52	(0)	30/35	(86)	12/12	(100)
Kenema	8/52	(15)	28/35	(80)	11/12	(92)
Kono	0/52	(0)	32/35	(91)	10/12	(83)
Koinadugu	0/52	(0)	30/35	(86)	6/12	(50)
Moyamba	0/52	(0)	31/35	(89)	7/12	(58)
Port Loko	0/52	(0)	29/35	(83)	12/12	(100)
Pujehun	0/52	(0)	31/35	(89)	12/12	(100)
Tonkolili	0/52	(0)	26/35	(74)	11/12	(92)
Western Area	0/52	(0)	31/35	(89)	12/12	(100)

^1^ Expected number of forms = Number of weeks in the study period. ^2^ Results for January-December 2019 published and available at [8]; ^3^ Results for March—October 2021 published and available at [10].

**Table 5:** Availability and consistency of reporting by Community Animal Health Workers (CAHWs) to the livestock disease surveillance program in Sierra Leone in January—March 2023.

District	Median Number (IQR ^1^) of CAHWs Available per District per Week	Percentage of CAHWs Reporting per Week		Number of Weeks with 100% Reporting from CAHWs/Expected Number of Reports (%)
		Minimum	Maximum	
All districts	10 (8–14)	11%	100%	88/180 (49%)
Bo	10 (10–10)	88%	100%	11/12 (92%)
Bombali	15 (15–15)	20%	100%	1/12 (8%)
Bonthe	7 (7–7)	57%	100%	2/12 (17%)
Falaba	9 (9–9)	100%	100%	12/12 (100%)
Kambia	13 (13–13)	46%	100%	1/12 (8%)
Kailahun	8 (7–8)	71%	100%	2/12 (17%)
Kerene	7 (7–10)	70%	100%	10/12 (83%)
Kenema	8 (8–8)	25%	100%	2/12 (17%)
Kono	15 (15–15)	60%	67%	0/12 (0%)
Koinadugu	14 (14–14)	86%	100%	2/12 (17%)
Moyamba	9 (9–9)	11%	78%	0/12 (0%)
Port Loko	10 (10–10)	100%	100%	12/12 (100%)
Pujehun	25 (25–26)	100%	100%	12/12 (100%)
Tonkolili	8 (8–10)	88%	100%	9/12 (75%)
Western Area	4 (4–4)	100%	100%	12/12 (100%)

^1^ IQR–Inter-quartile range.

**Table 6:** Changes in data quality issues and shortfalls in reporting formats used to submit Animal Health Weekly Reporting forms by district-level officers in Sierra Leone in 2021 versus 2023.

Gaps Identified in 2021 *	Status in 2023
Data quality issues found in 152 out of 1950 sub-reports	Data quality issues found in 7 out of 1012 sub-reports
Inconsistency between numbers of susceptible vs. sick vs. treated (14 sub-reports)	No inconsistency
Antimicrobial/anthelmintic use was reported, but the list of drugs prescribed did not include any antimicrobial/anthelmintic (97 sub-reports)	No inconsistency
Treatment details provided include antimicrobials/anthelmintic drugs, but the number treated with antimicrobials//anthelmintics reported as “0” (27 sub-reports)	One sub-report
Missing data in sub-reports	Seven sub-reports
Diagnosis missing, though treatment details and numbers of sick animals are provided (22 sub-reports)	No inconsistency
Name of affected species missing (22 sub-reports)	One sub-report
Humans reported as affected species (11 sub-reports) in cases of dog and monkey bites, with no information on the treatment offered to affected animal species	Six sub-reports
Limitations in the design of reporting fields	
No uniform categorizations for locations, diseases, species, and treatment (use of free text fields) Specific name of antimicrobial was not mentioned, and therefore antimicrobial use classification categorization was not possible (253 sub-reports)	Issue persists Issue persists (120 sub-reports)
Unable to ascertain if the disease reports are from a single farm or multiple farms	Issue persists
Anthelmintics are also reported under usage of antimicrobials	Anthelmintics reported separately
Parameters missing in the current format	
Timeliness of reporting	Not available
Number of CAHWs reporting per week	Reported
Level of diagnostic certainty	Not available
Duration of treatment and route of administration of drugs	Not available
Data on follow-up of cases	Not available
Clear disaggregation for sub-reports	Not available

* results for March—October 2021 published and available at [10].

**Table 7:** Number of livestock treated with antimicrobial drugs by animal species in Sierra Leone during January—March 2023 compared to March—October 2021.

Livestock Species (as Reported)	March—October 2021 ^1^					January—March 2023				
	Susceptible Livestock	Sick Animals		Sick Animals Treated with Antimicrobials		Susceptible Livestock	Sick Animals		Sick Animals Treated with Antimicrobials	
	**N**	**N**	**(%) ^2^**	**n**	**(%) ^3^**	**N**	**n**	**(%) ^2^**	**n**	**(%) ^3^**
Cattle	1175	362	(30.8)	168	(46.4)	460	89	(19)	35	(39)
Dogs	711	117	(16.5)	43	(36.8)	218	93	(43)	12	(13)
Donkeys	7	4	(57.1)	1	(25.0)	0		0	0	0
Fowl	6569	914	(13.9)	3	(0.3)	1245	101	(8)	8	(8)
Goat	22,198	6229	(28.1)	1576	(25.3)	15306	2911	(19)	799	(27)
Goats and Sheep ^4^	1409	835	(59.3)	402	(48.1)	34	17	(50)	0	0
Horse	40	16	(40.0)	15	(93.8)	0		0	0	0
Pig	1930	574	(29.7)	105	(18.3)	649	316	(49)	98	(31)
Rabbit	52	30	(57.7)	4	(13.3)	85	13	(15)	0	0
Sheep	10,775	2691	(25.0)	692	(25.7)	16,471	2326	(14)	495	(21)
Not recorded	401	111	(27.6)	11	(2.7)	17	3	(18)	3	(100)
Total	45,267	11,883	(26.2)	3020	(25.4)	34,485	5869	(17)	1450	(25)

^1^ Reports with discrepancies between numbers of susceptible vs. sick vs. treated were excluded from analysis; ^2^ Percentage of sick out of susceptible animals; ^3^ Percentage of livestock initiated on antimicrobial or anthelmintic treatment out of sick animals; ^4^ Some reports mentioned species as “Goats and sheep”, and disaggregated species wise information was not available, hence retained as a separate category. Reference: Results for March—October 2021 published and available at [10].

**Table 8:** Antimicrobial use based on WOAH list of antimicrobial agents of veterinary importance in Sierra Leone during January—March 2023 compared to March—October 2021.

Livestock Species (as Reported)	March—October 2021			January—March 2023		
	Any Antimicrobial Used	Critically Important Antimicrobials for Veterinary Use		Any Antimicrobial Used	Critically Important Antimicrobials for Veterinary Use ^1^	
	N	N	(%) ^2^	N	N	(%) ^2^
Cattle	168	145	(86.3)	35	13	(37.1)
Dogs	43	37	(86.0)	12	6	(50)
Donkeys	1	0	(0.0)	0	0	0
Fowl	3	3	(100.0)	8	0	0
Goat	1576	1070	(67.9)	799	537	(67.2)
Goats and Sheep ^3^	402	402	(100.0)	0	0	0
Horse	15	15	(100.0)	0	0	0
Pig	105	89	(84.8)	98	59	(60.2)
Rabbit	4	4	(100.0)	0	0	0
Sheep	692	557	(80.5)	495	376	(76)
Not recorded	11	3	(27.3)	3	0	0
Total	3020	2325	(76.9)	1450	994	(68.6)

^1^ The critically important antimicrobials that were most commonly being used were Tylosin, Oxytetracycline, Kenflox (Levofloxacin), Trimethoprim + Sulfonamide (TMPS), Penstrep (penicillin-streptomycin), and gentamicin. ^2^ Percentages calculated out of all livestock initiated on antimicrobial treatment. ^3^ Some reports mentioned species as “Goats and sheep”, and disaggregated species-wise information was not available, hence retained as a separate category. Reference: Results for March- October 2021 published and available at [10].

## Data Availability

Requests to access these data can be sent to the corresponding author.

## Supplementary Materials

The following supporting information can be downloaded at: https://www.mdpi.com/article/10.3390/tropicalmed8080408/s1, File S1: Community Base Surveillance; S2: Animal health weekly reporting format; P1: Three-minute lightening power point presentation; P2: Full presentation; H1 = Handout.
