# Supplementary material for: Improvement in the Surveillance System for Livestock Diseases and Antimicrobial Use Following Operational Research Studies in Sierra Leone January–March 2023
Source: Trop Med Infect Dis. 2023 Aug 10;8(8):408. doi: 10.3390/tropicalmed8080408 (PMC10459562; doi:10.3390/tropicalmed8080408)
Supplement: Supplementary file 1 [file tropicalmed-08-00408-s001.zip › H1.pdf]

Brief summary

May 2022, Volume 1, Number 1

Livestock disease surveillance in Sierra Leone: Significant improvement but gaps remain in data quality and use of antimicrobials

[1]Reference: Bangura, F.I.; Leno, A.; Hann, K.; Timire, C.; Nair, D.; Bah, M.A.; Gborie, S.R.; Satyanarayana, S.; Edwards, J.K.; Davtyan, H.; et al. An Update on the Surveillance of Livestock Diseases and Antimicrobial Use in Sierra Leone in 2021—An Operational Research Study. *Int. J. Environ. Res. Public Health* **2022**, *19*, 5294. https://doi.org/10.3390/ijerph19095294

e-mail: turaybangfatmataisatu@gmail.com

Key Messages

- **Significant improvement in the animal health surveillance system –** Weekly reports available from all 15 agricultural districts (2021) vs only three districts (2016 -2019)
- **Gaps remain** in quality of data reported through surveillance.
- ~**25% of the livestock had an infectious disease**, all of them diagnosed without laboratory confirmation.
- **A quarter of the sick animals had received an antimicrobial drug**. Most animals received World Organization for Animal Health's **"veterinary critically important antimicrobials" (77%)** and World Health Organization's "critically" (17%) and "highly important" (60%) antimicrobials for human health.
- **Enhanced antimicrobial stewardship** (setting standards for diagnosis of livestock diseases and antimicrobial use, strengthening recording and reporting system, capacity building of field staff) is urgently needed to prevent misuse of antimicrobials that are of significance in animal and human health.

What is the problem and why is it important?

- Consumption of antimicrobials in animals, particularly in livestock, is increasing at an unprecedented pace → Rising levels of anti-antimicrobial drug resistance (AMR)→ Potential for catastrophic effects on human health
- **Deaths linked to AMR are highest in Western African countries,** such as Sierra Leone compared to other regions globally
- Livestock are major source of livelihood in predominantly agrarian Sierra Leone
- **Sierra Leone's National Strategic Plan for Combatting Antimicrobial Resistance** (2018-22) advocates for the **surveillance of livestock diseases, judicious use and monitoring of antimicrobial agents** used in the treatment of livestock diseases.
- Previous operational research study (2019): Routine **reporting of livestock disease and the use of antimicrobials was very poor in the country.**
- In response to the previous study's findings, staff at all 15 district livestock offices were trained on the routine recording and reporting systems on livestock diseases and antimicrobial use.

Parameters missing in the current reporting format:
- Timeliness of reporting
- Level of diagnostic certainty
- Duration of treatment and route of administration of drugs
- Treatment outcomes

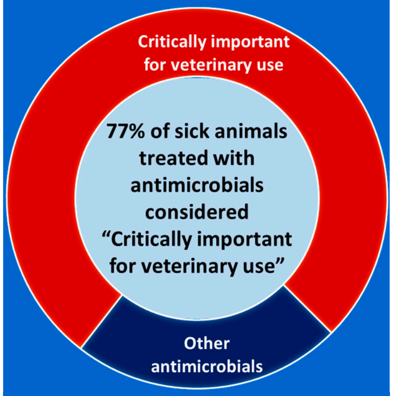

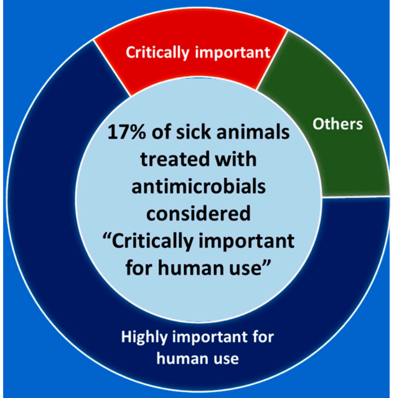

Critically important antimicrobials: Essential for treating certain diseases in livestock/humans **May not have substitutes** if resistance emerges

How did we measure it?

- Analysis of **weekly surveillance reports from the 15 district livestock offices during the period March- October 2021**, data used to describe livestock diseases and antimicrobials utilized in their treatment
- Designed as a **follow up of previous operational research study**

What did we find?

- **461 (88%) out of an expected 525 weekly reports for the were available** from 15 agricultural districts over the 35-week study period
- Reporting: Highest from Bombali district (100%), lowest from Tonkolili district (74%)
- Certain **gaps remain in the reporting system:**
  - Non uniform terminology for reporting diseases and antimicrobials
  - Lack of certain data elements required for monitoring purposes (for e.g., treatment outcomes)
  - Assessment of performance of Community Animal Health Workers was not possible
- **25% of the livestock had an infectious disease;** none were confirmed in a microbiological laboratory
- **Use of antimicrobials:**
  - 25% of the sick animals had received an antimicrobial drug
  - 77% of sick animals received "Veterinary critically important" category antimicrobials (World Organization for Animal Health (OIE) classification)

Implications

- Ministry of Agriculture should take a lead on **further improving reporting system** to generate actionable data on disease burden, antimicrobial use, and resistance:
  - Consultations with experts and related ministries
  - Development of standard reporting formats with uniform terminology
  - Inclusion of left-over monitoring indicators in reporting formats
  - Dedicated human resources for ensuring timely review and analysis of reports
- **Urgent need for enhanced antimicrobial stewardship** in Livestock and Veterinary Services Division to prevent misuse of antimicrobials:
  - Ensure adherence to standard guidelines on antimicrobial use
  - Capacity building of livestock officers and community workers
  - Strengthen the laboratory infrastructure for microbiological confirmation of livestock diseases
