# Supplementary material for: Improvement in the Surveillance System for Livestock Diseases and Antimicrobial Use Following Operational Research Studies in Sierra Leone January–March 2023
Source: Trop Med Infect Dis. 2023 Aug 10;8(8):408. doi: 10.3390/tropicalmed8080408 (PMC10459562; doi:10.3390/tropicalmed8080408)
Supplement: Supplementary file 1 [file tropicalmed-08-00408-s001.zip › P1.pptx]

## Slide 1
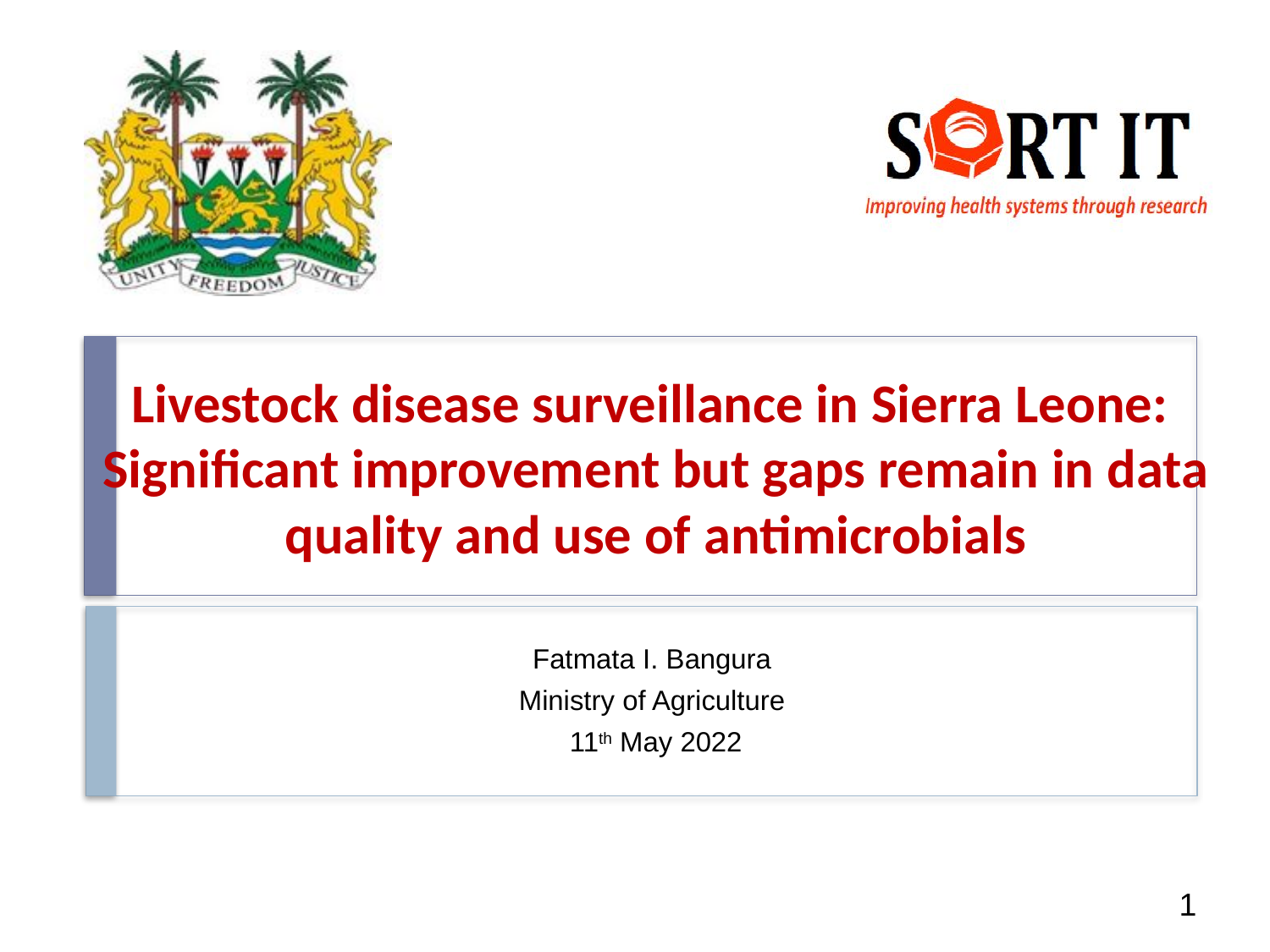

# Livestock disease surveillance in Sierra Leone: Significant improvement but gaps remain in data quality and use of antimicrobials
Fatmata I. Bangura
Ministry of Agriculture
11th May 2022
1

## Slide 2
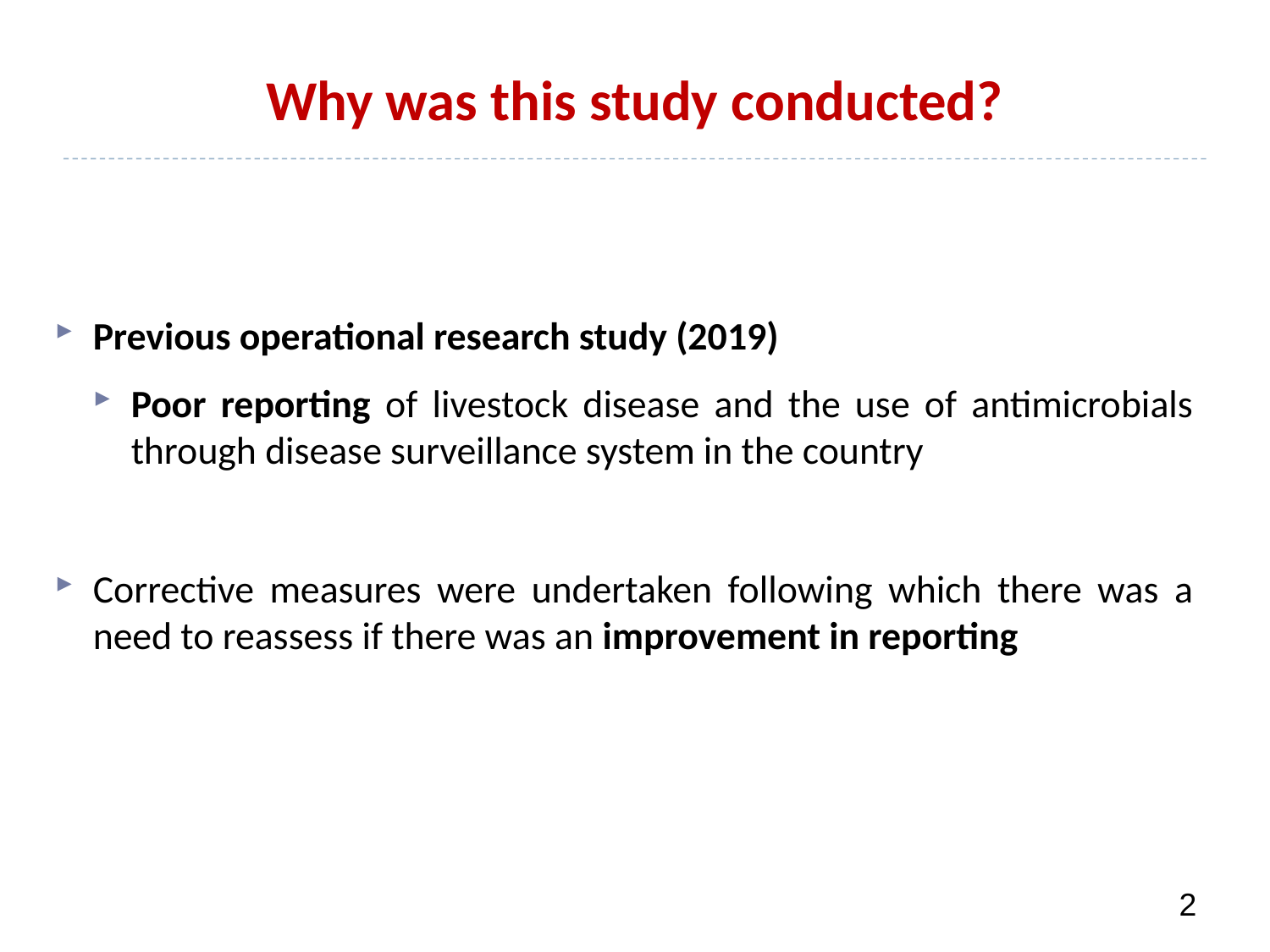

# Why was this study conducted?
Previous operational research study (2019)
Poor reporting of livestock disease and the use of antimicrobials through disease surveillance system in the country
Corrective measures were undertaken following which there was a need to reassess if there was an improvement in reporting
2

## Slide 3
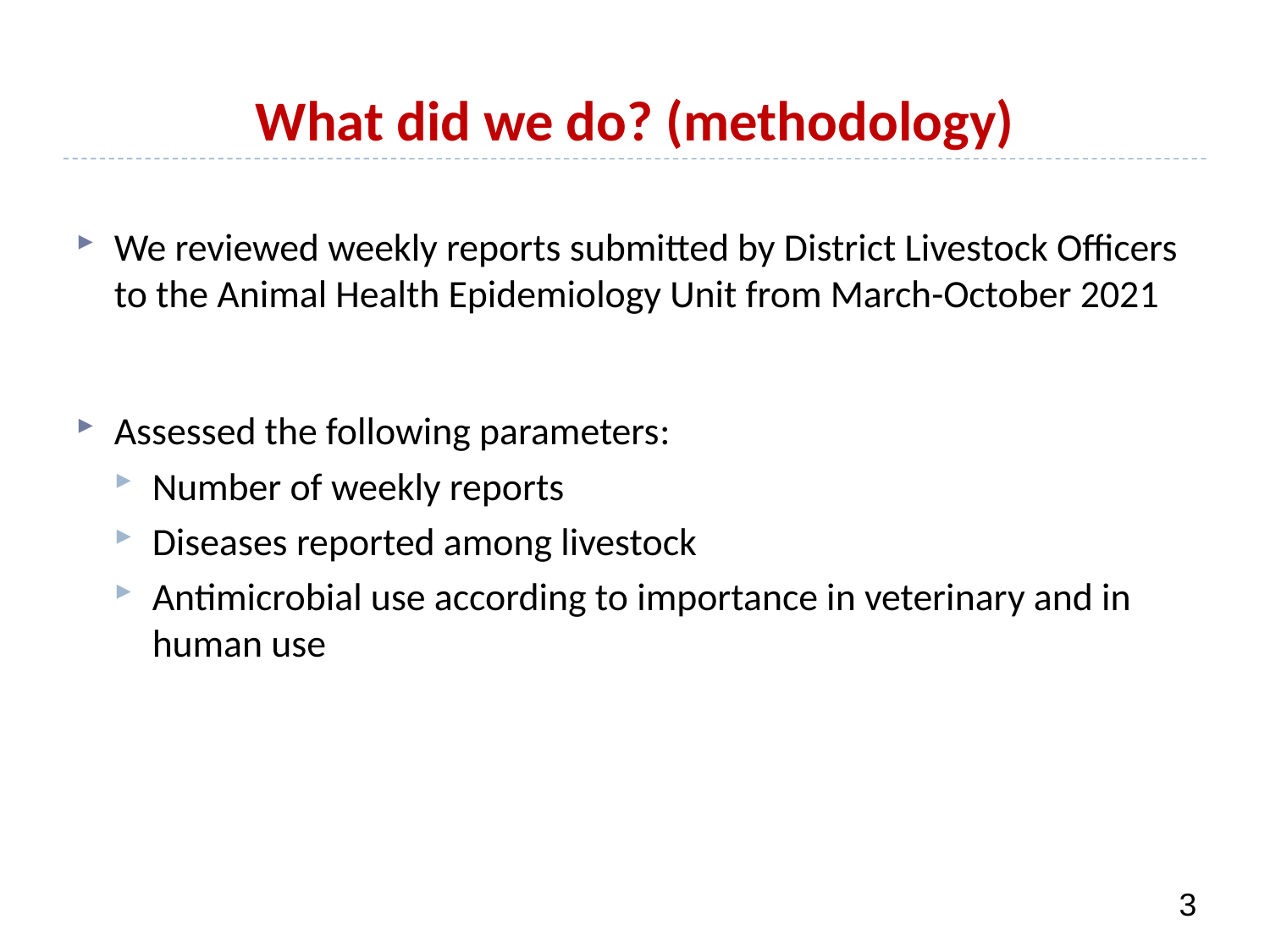

# What did we do? (methodology)
We reviewed weekly reports submitted by District Livestock Officers to the Animal Health Epidemiology Unit from March-October 2021
Assessed the following parameters:
Number of weekly reports
Diseases reported among livestock
Antimicrobial use according to importance in veterinary and in human use
3

## Slide 4
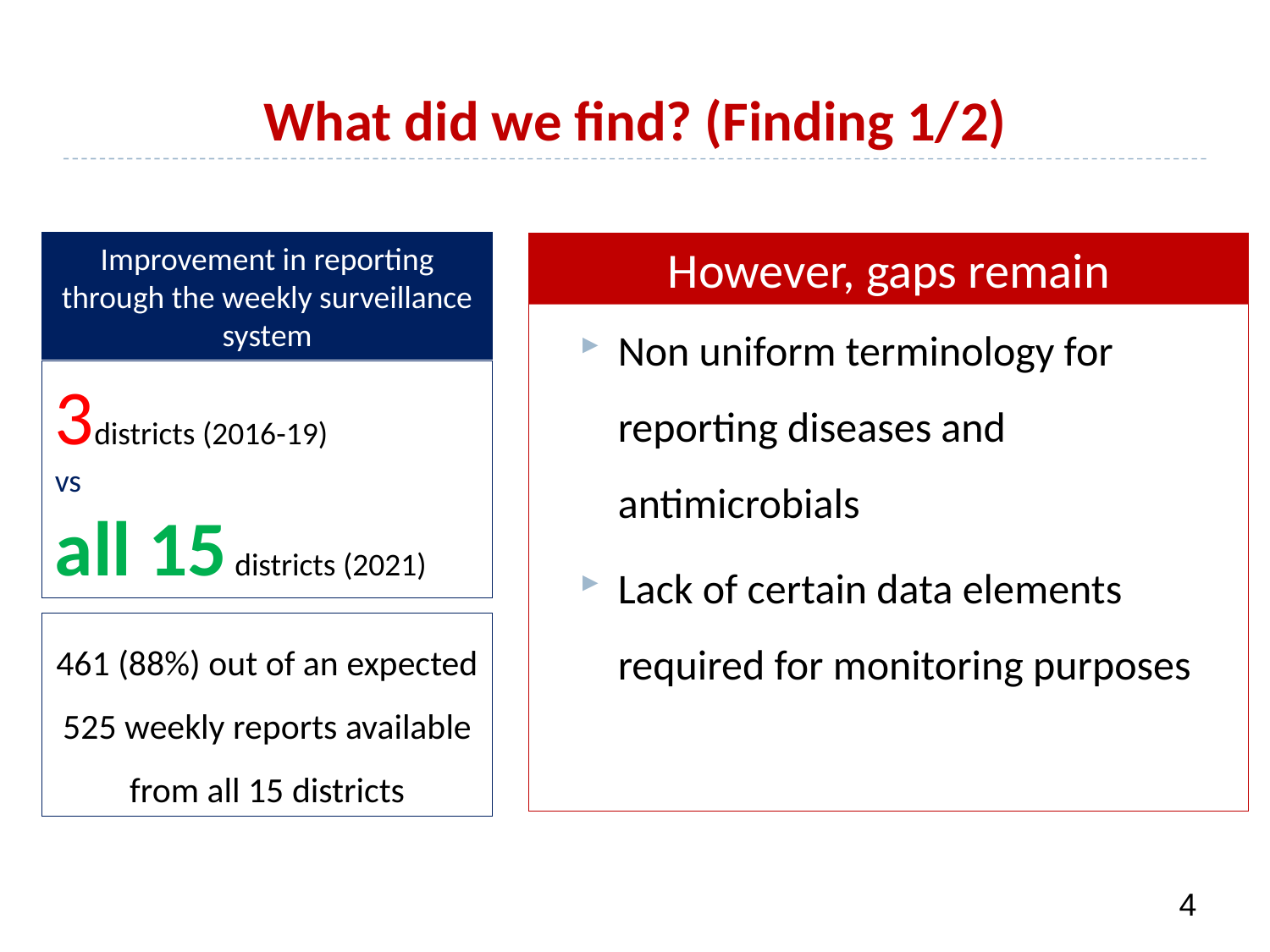

# What did we find? (Finding 1/2)
Improvement in reporting through the weekly surveillance system
Non uniform terminology for reporting diseases and antimicrobials
Lack of certain data elements required for monitoring purposes
However, gaps remain
3districts (2016-19)
vs
all 15 districts (2021)
461 (88%) out of an expected 525 weekly reports available from all 15 districts
4

## Slide 5
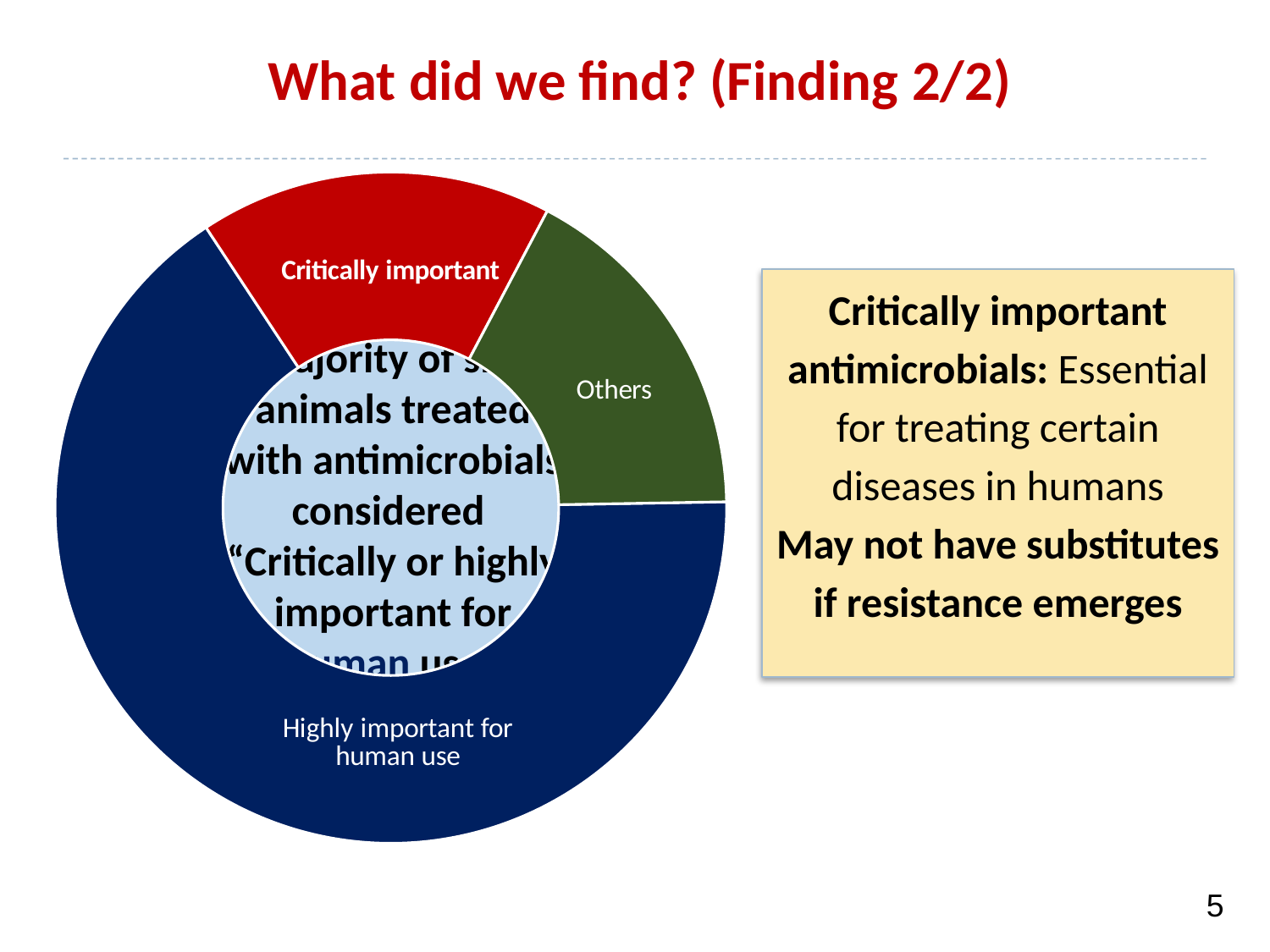

# What did we find? (Finding 2/2)
### Chart
| Category | Sales |
|---|---|
| Highly important for human use | 66.0 |
| Critically important | 17.0 |
| Others | 17.0 |Majority of sick animals treated with antimicrobials considered
“Critically or highly important for human use”
Critically important antimicrobials: Essential for treating certain diseases in humans
May not have substitutes if resistance emerges
5

## Slide 6
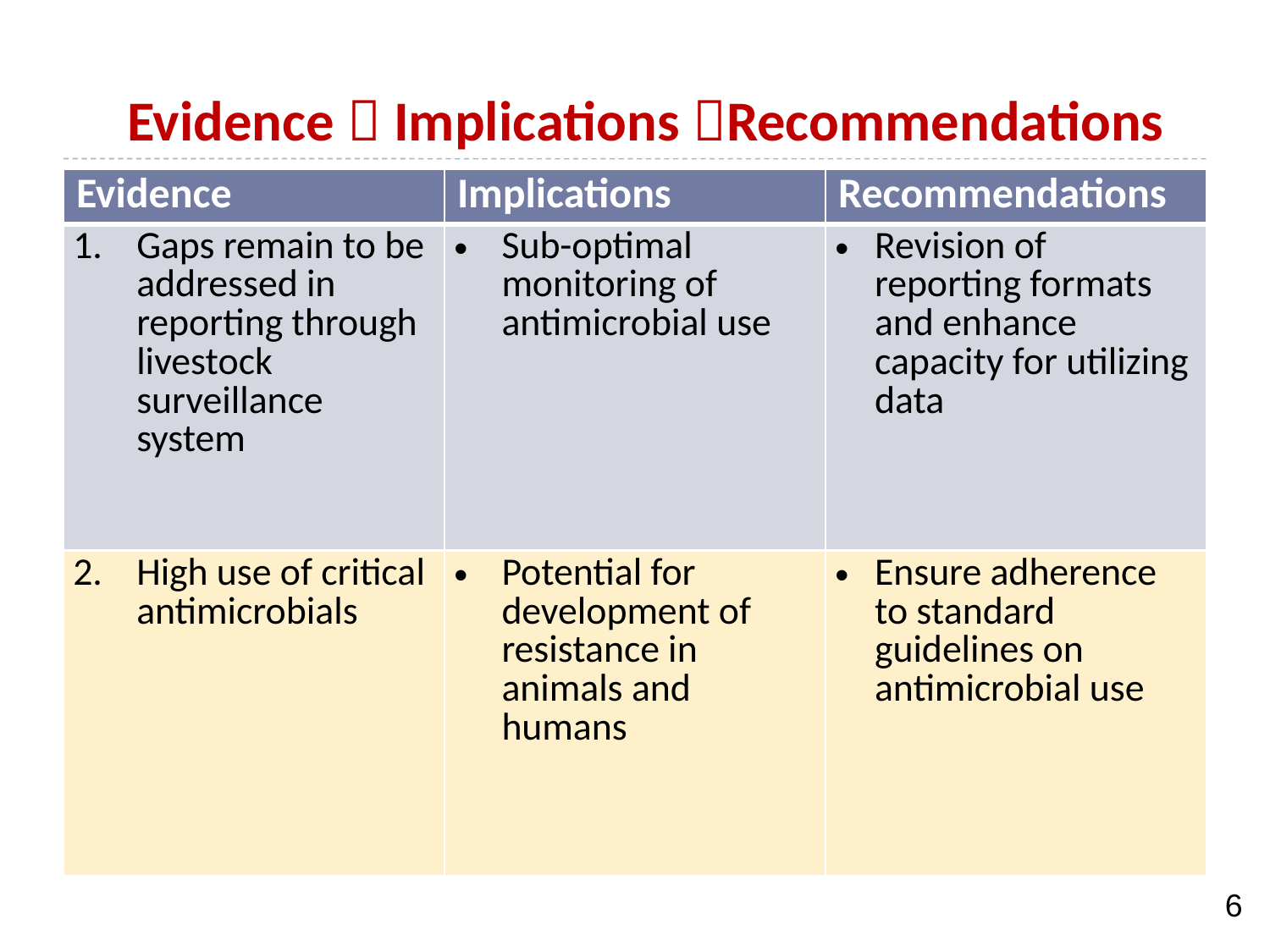

# Evidence  Implications Recommendations
| Evidence | Implications | Recommendations |
| --- | --- | --- |
| Gaps remain to be addressed in reporting through livestock surveillance system | Sub-optimal monitoring of antimicrobial use | Revision of reporting formats and enhance capacity for utilizing data |
| High use of critical antimicrobials | Potential for development of resistance in animals and humans | Ensure adherence to standard guidelines on antimicrobial use |
| --- | --- | --- |
7
6

## Slide 7
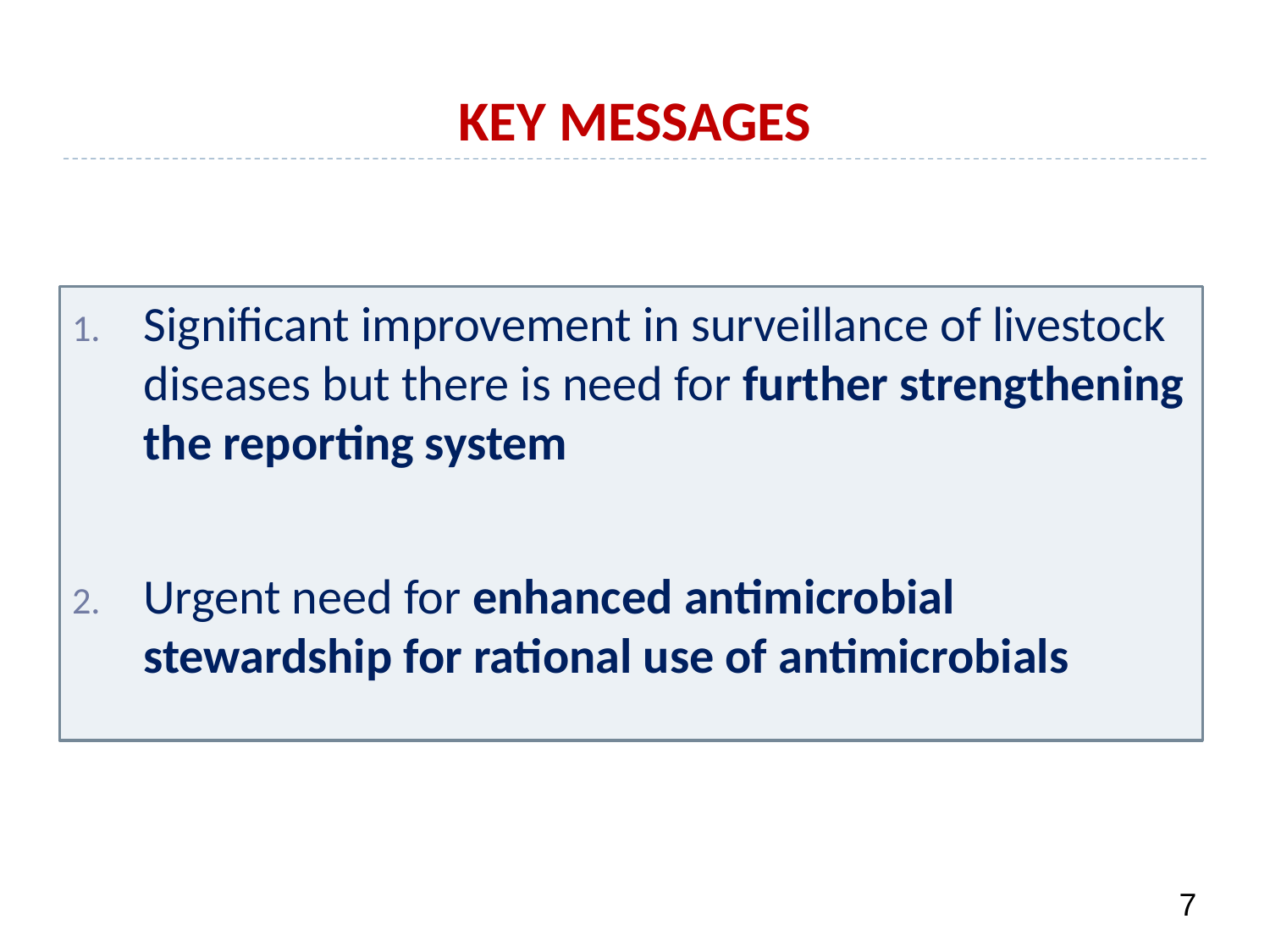

# KEY MESSAGES
Significant improvement in surveillance of livestock diseases but there is need for further strengthening the reporting system
Urgent need for enhanced antimicrobial stewardship for rational use of antimicrobials
7

## Slide 8
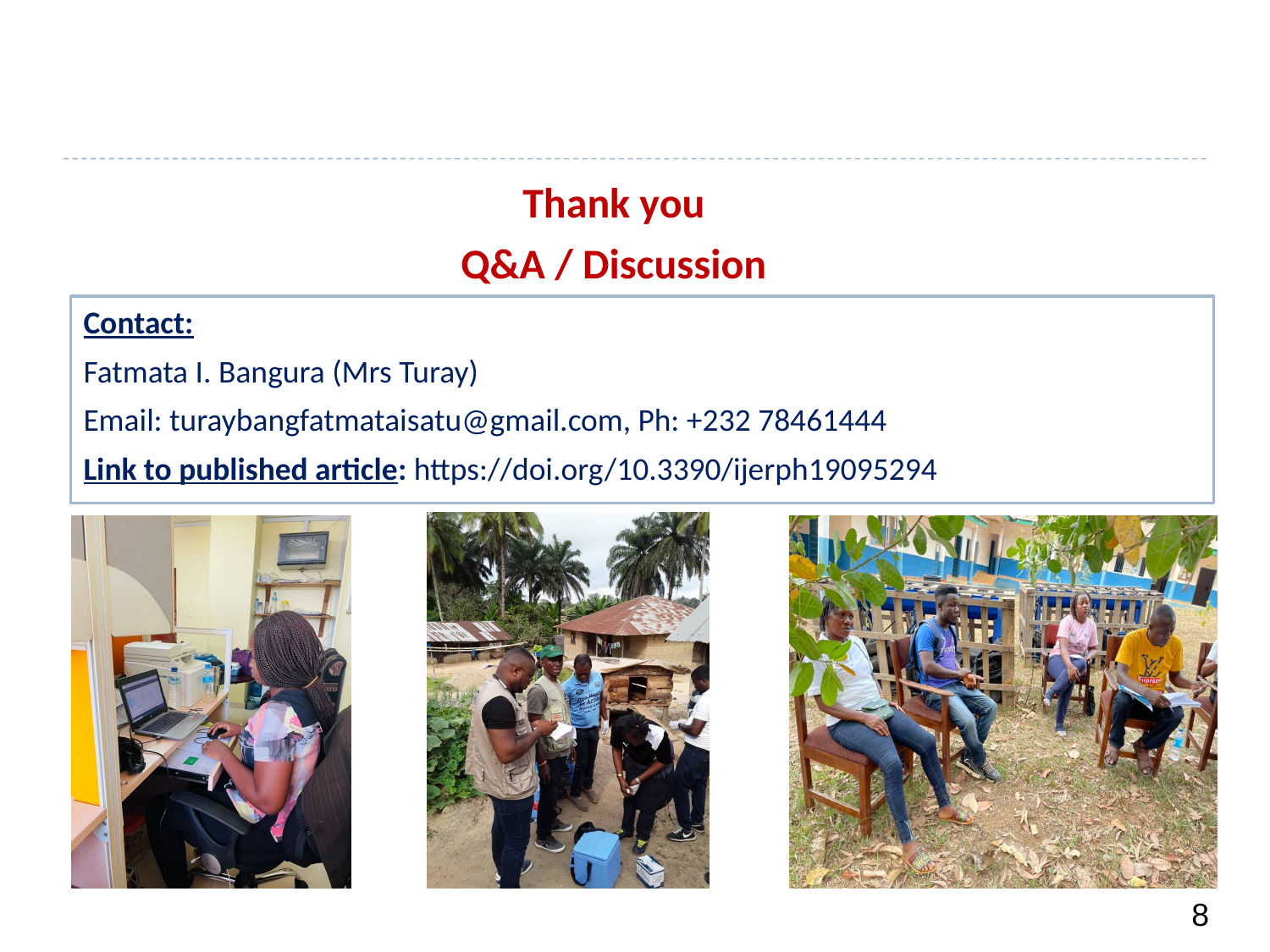

Thank you
Q&A / Discussion
Contact:
Fatmata I. Bangura (Mrs Turay)
Email: turaybangfatmataisatu@gmail.com, Ph: +232 78461444
Link to published article: https://doi.org/10.3390/ijerph19095294
8
