# Supplementary material for: Improvement in the Surveillance System for Livestock Diseases and Antimicrobial Use Following Operational Research Studies in Sierra Leone January–March 2023
Source: Trop Med Infect Dis. 2023 Aug 10;8(8):408. doi: 10.3390/tropicalmed8080408 (PMC10459562; doi:10.3390/tropicalmed8080408)
Supplement: Supplementary file 1 [file tropicalmed-08-00408-s001.zip › P2.pptx]

## Slide 1
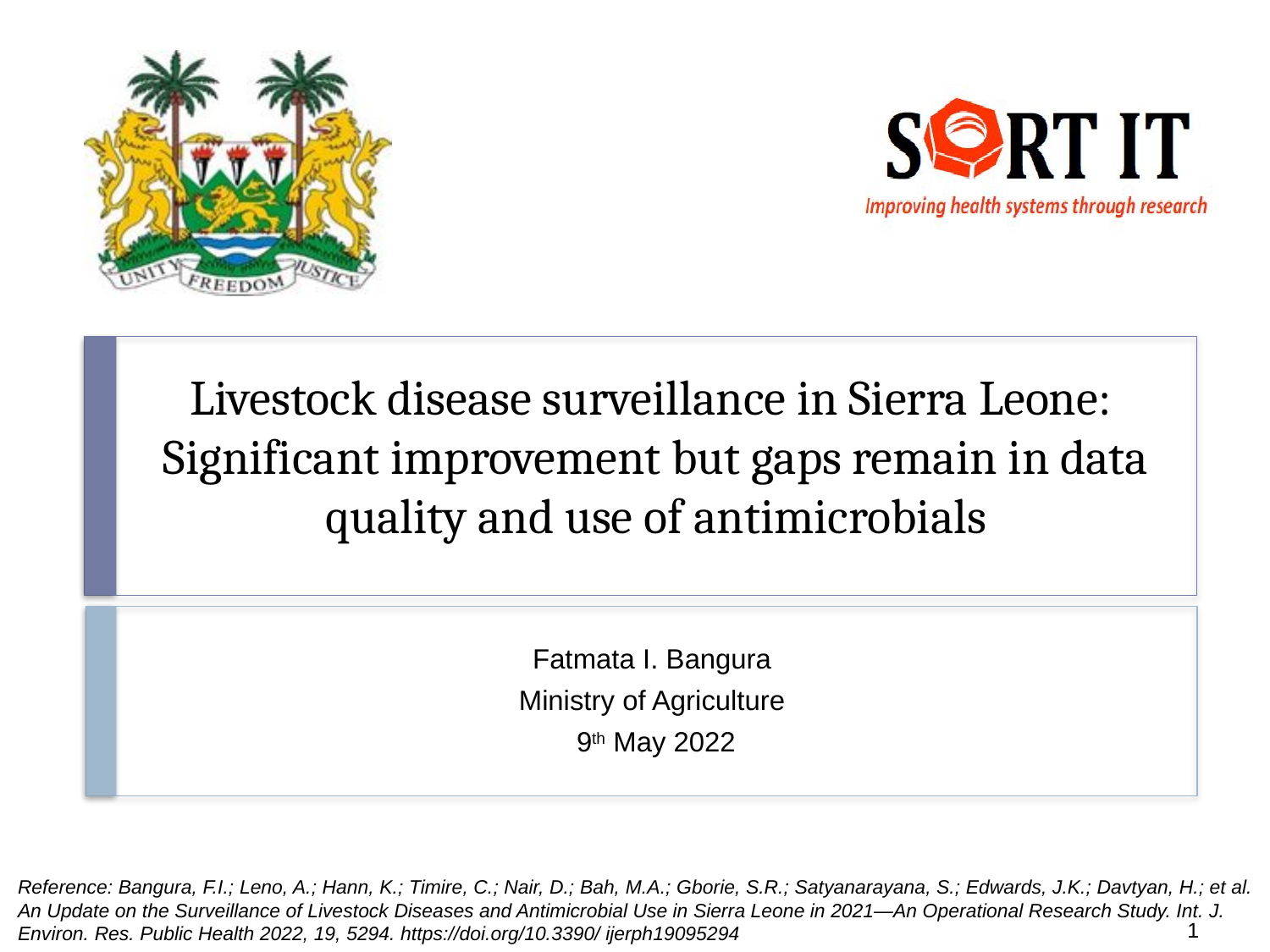

# Livestock disease surveillance in Sierra Leone: Significant improvement but gaps remain in data quality and use of antimicrobials
Fatmata I. Bangura
Ministry of Agriculture
9th May 2022
Reference: Bangura, F.I.; Leno, A.; Hann, K.; Timire, C.; Nair, D.; Bah, M.A.; Gborie, S.R.; Satyanarayana, S.; Edwards, J.K.; Davtyan, H.; et al. An Update on the Surveillance of Livestock Diseases and Antimicrobial Use in Sierra Leone in 2021—An Operational Research Study. Int. J. Environ. Res. Public Health 2022, 19, 5294. https://doi.org/10.3390/ ijerph19095294
1

## Slide 2
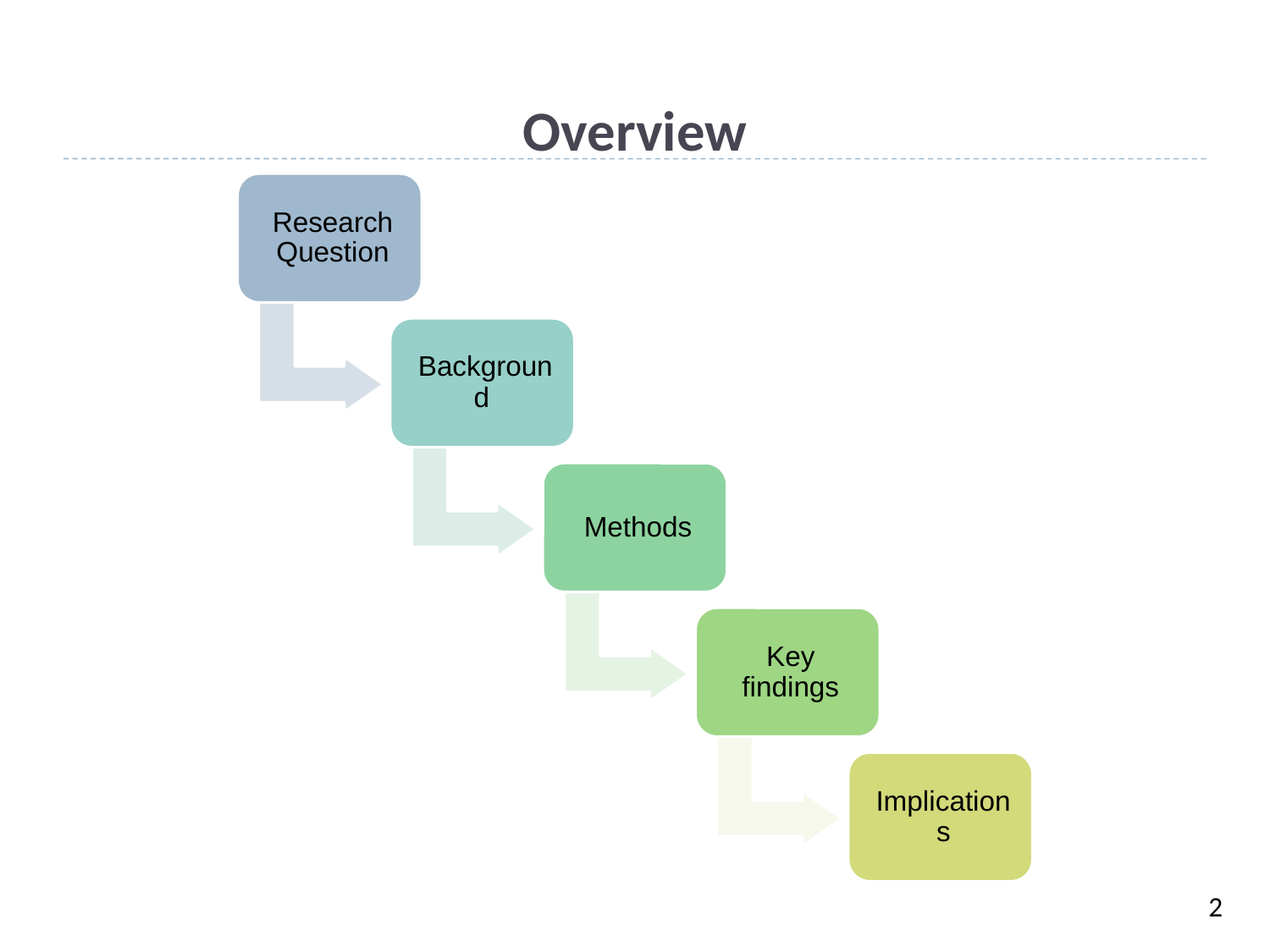

# Overview
2

## Slide 3
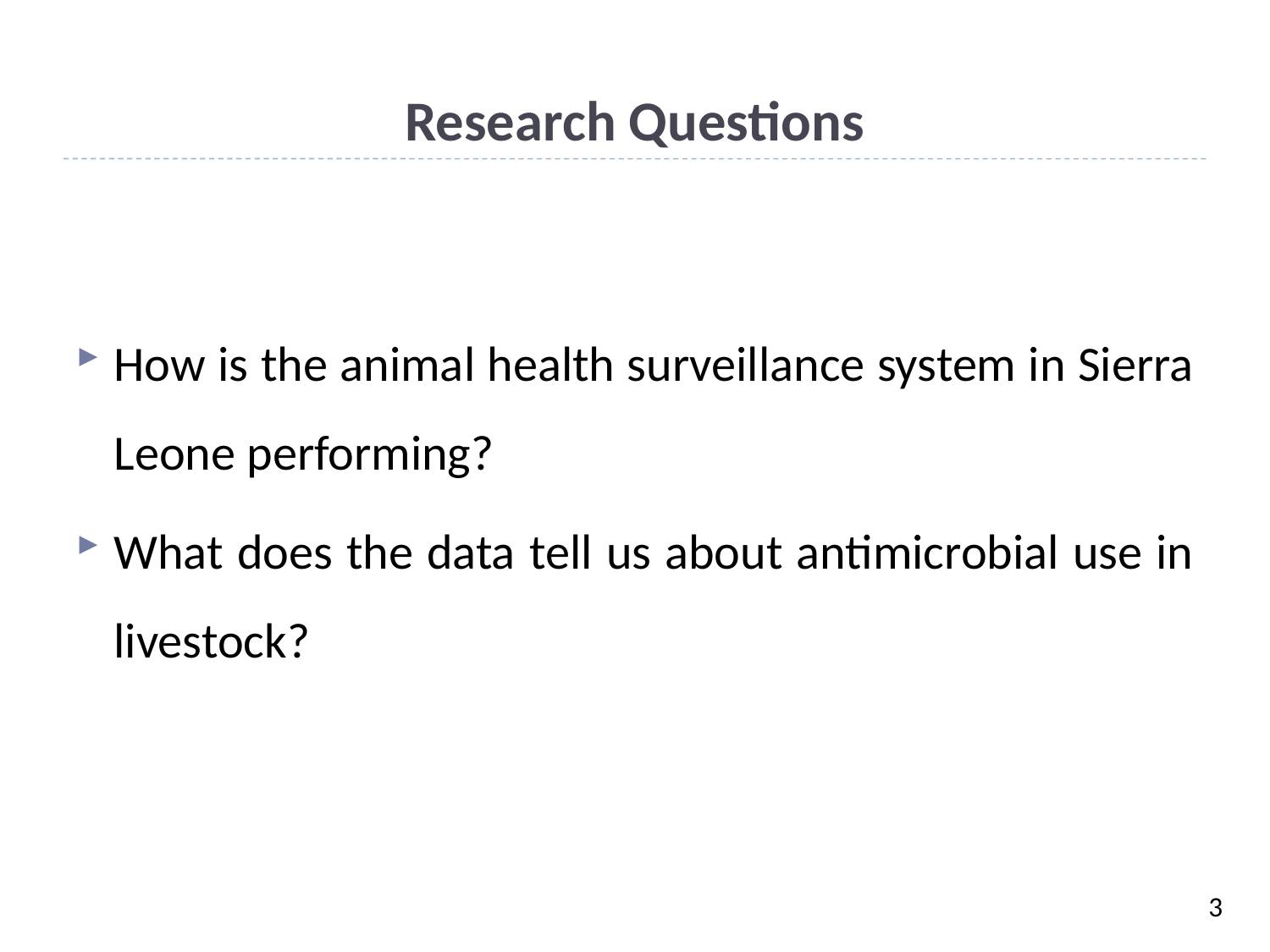

# Research Questions
How is the animal health surveillance system in Sierra Leone performing?
What does the data tell us about antimicrobial use in livestock?
3

## Slide 4
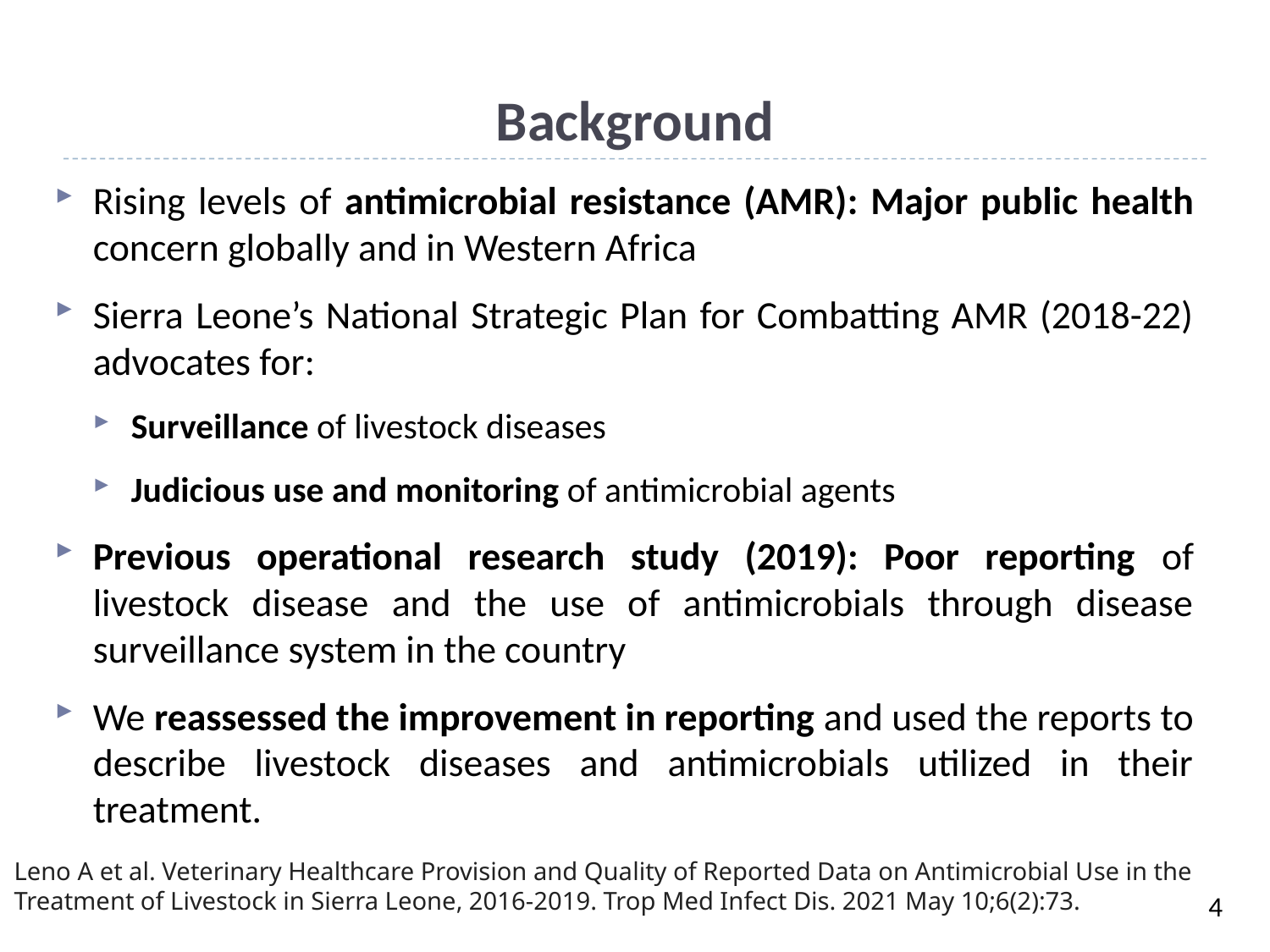

# Background
Rising levels of antimicrobial resistance (AMR): Major public health concern globally and in Western Africa
Sierra Leone’s National Strategic Plan for Combatting AMR (2018-22) advocates for:
Surveillance of livestock diseases
Judicious use and monitoring of antimicrobial agents
Previous operational research study (2019): Poor reporting of livestock disease and the use of antimicrobials through disease surveillance system in the country
We reassessed the improvement in reporting and used the reports to describe livestock diseases and antimicrobials utilized in their treatment.
Leno A et al. Veterinary Healthcare Provision and Quality of Reported Data on Antimicrobial Use in the Treatment of Livestock in Sierra Leone, 2016-2019. Trop Med Infect Dis. 2021 May 10;6(2):73.
4

## Slide 5
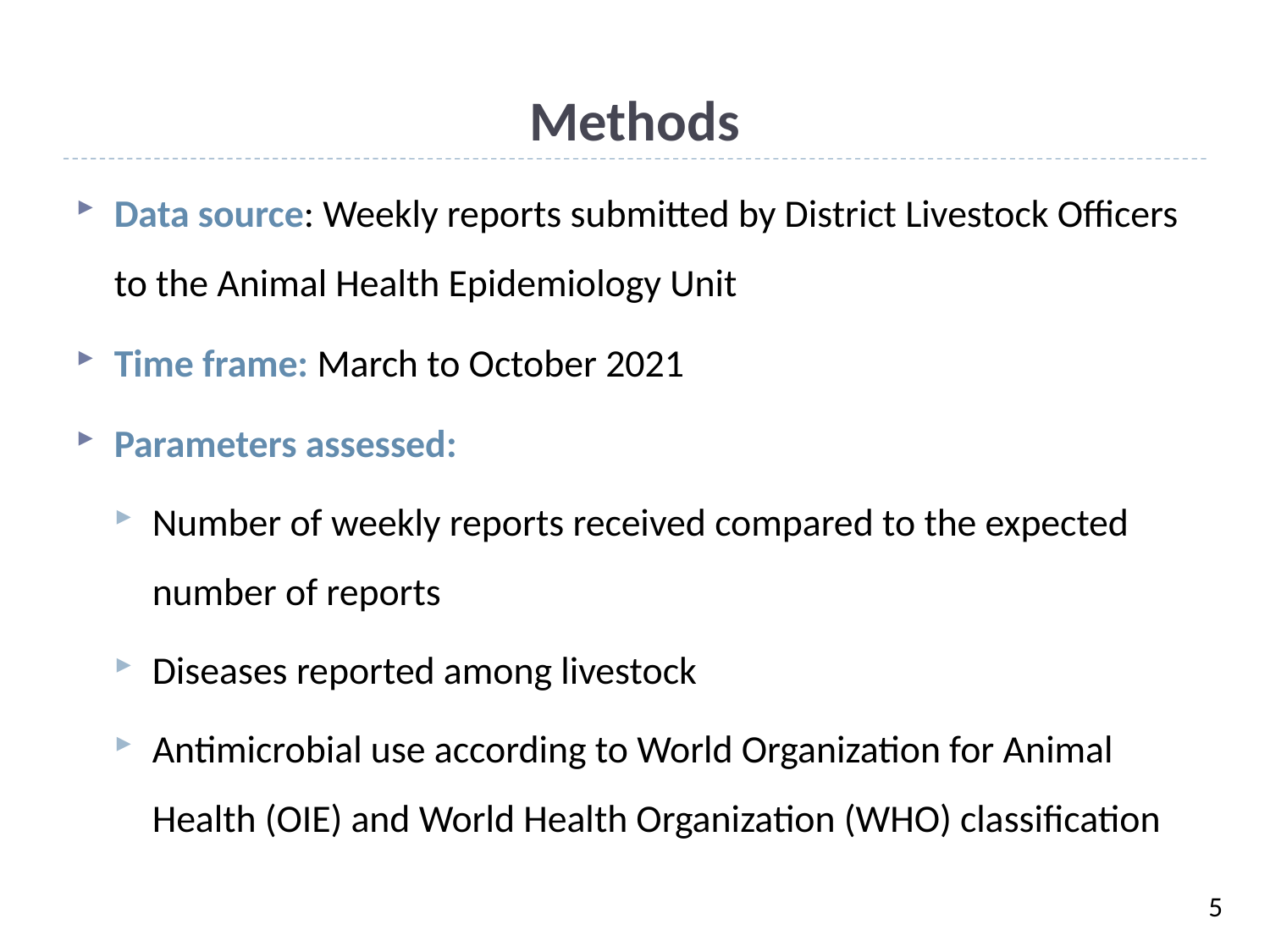

# Methods
Data source: Weekly reports submitted by District Livestock Officers to the Animal Health Epidemiology Unit
Time frame: March to October 2021
Parameters assessed:
Number of weekly reports received compared to the expected number of reports
Diseases reported among livestock
Antimicrobial use according to World Organization for Animal Health (OIE) and World Health Organization (WHO) classification
5

## Slide 6
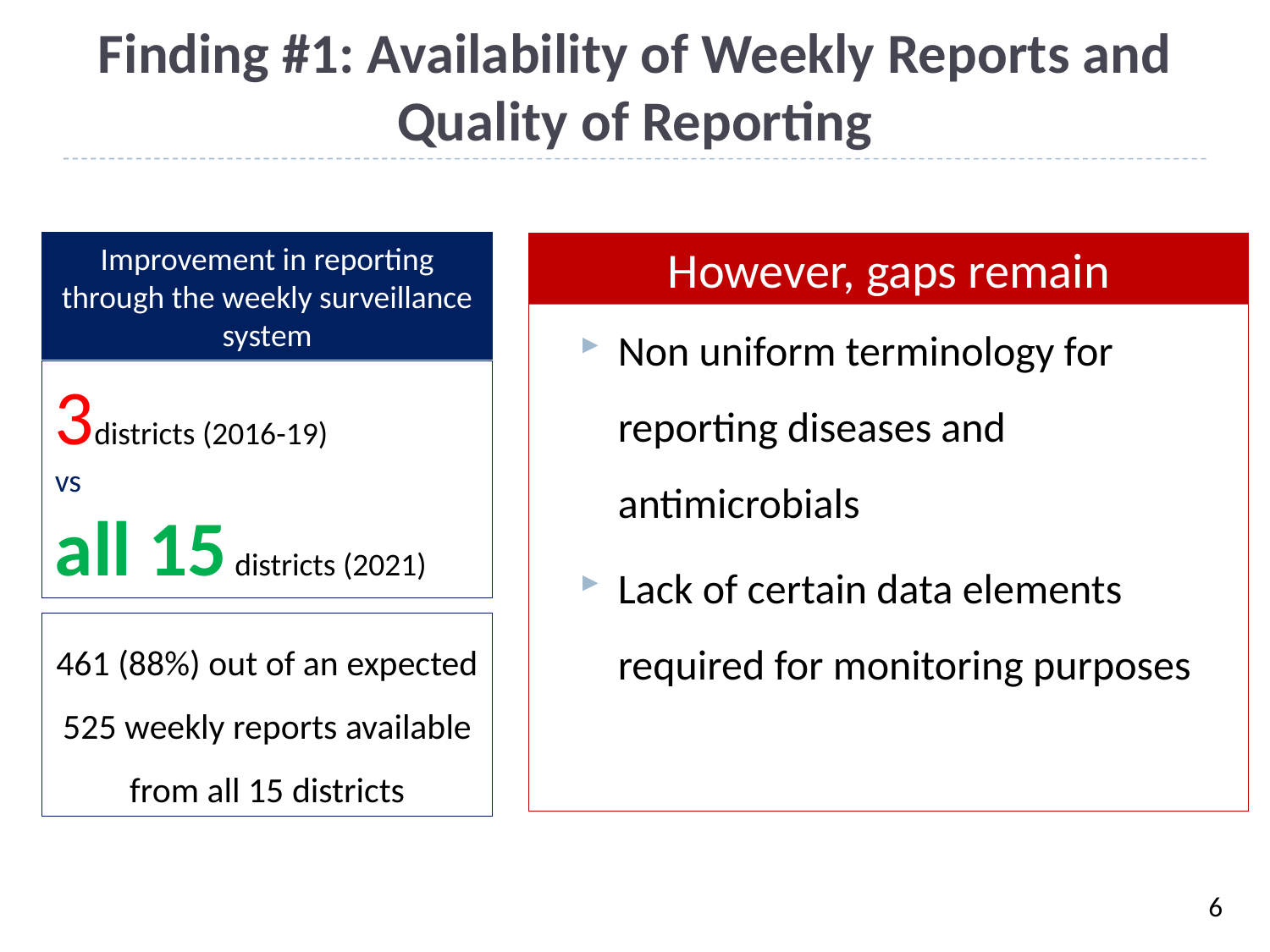

# Finding #1: Availability of Weekly Reports and Quality of Reporting
Improvement in reporting through the weekly surveillance system
Non uniform terminology for reporting diseases and antimicrobials
Lack of certain data elements required for monitoring purposes
However, gaps remain
3districts (2016-19)
vs
all 15 districts (2021)
461 (88%) out of an expected 525 weekly reports available from all 15 districts
6

## Slide 7
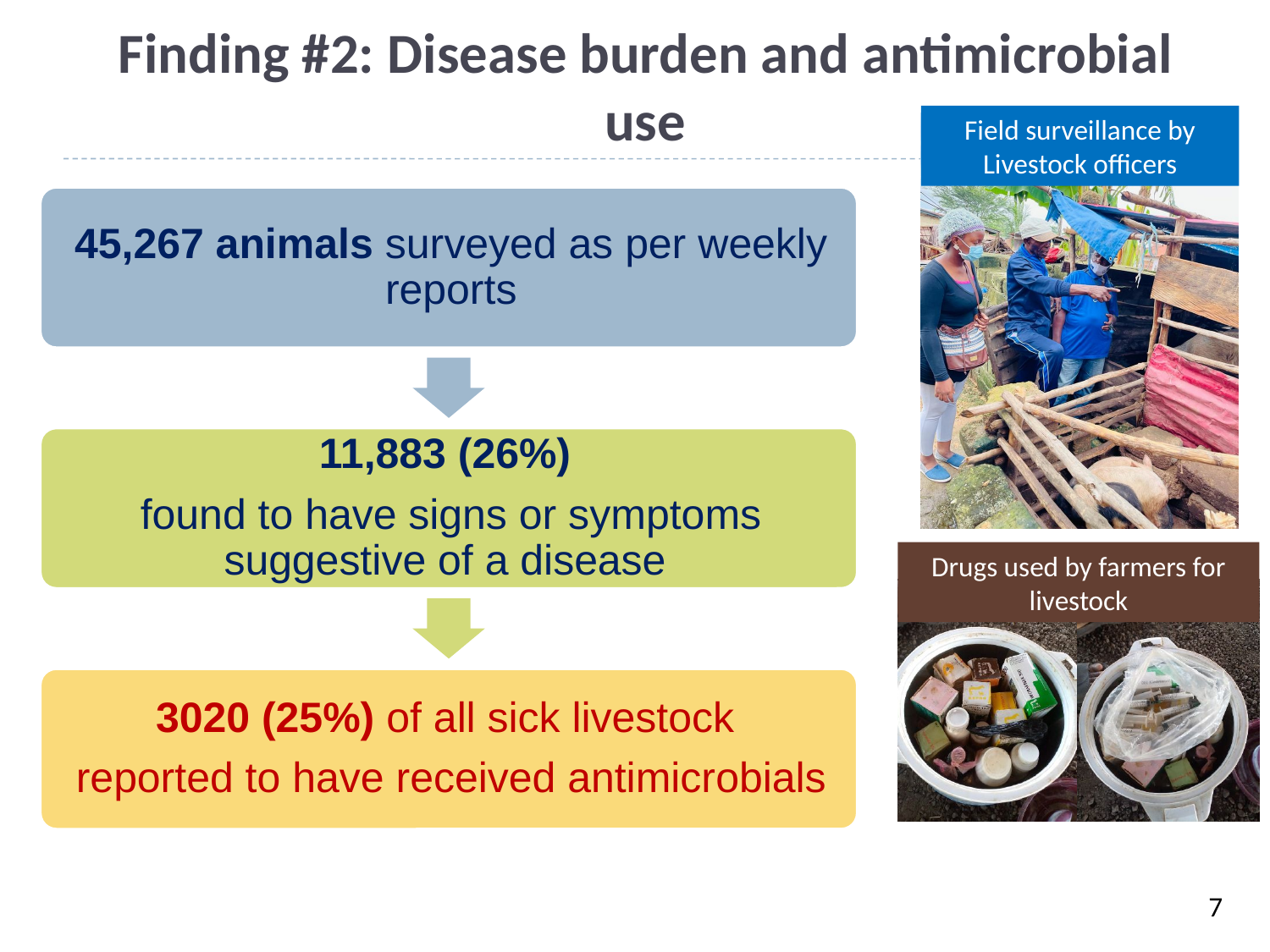

# Finding #2: Disease burden and antimicrobial use
Field surveillance by Livestock officers
Drugs used by farmers for livestock
7

## Slide 8
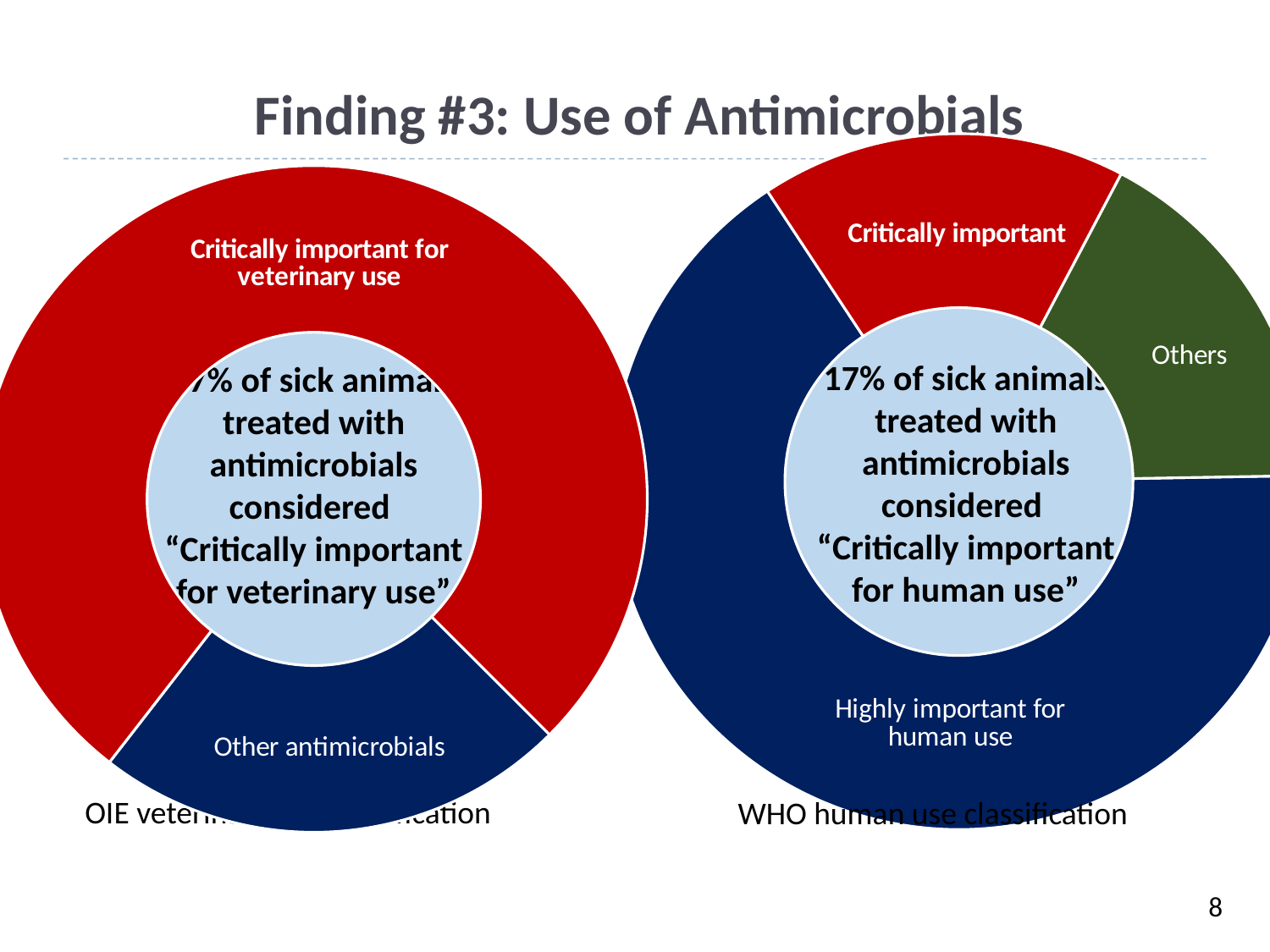

# Finding #3: Use of Antimicrobials
### Chart
| Category | Sales |
|---|---|
| Highly important for human use | 66.0 |
| Critically important | 17.0 |
| Others | 17.0 |
### Chart
| Category | Sales |
|---|---|
| Other antimicrobials | 23.0 |
| Critically important for veterinary use | 77.0 |17% of sick animals treated with antimicrobials considered
“Critically important for human use”
77% of sick animals treated with antimicrobials considered
“Critically important for veterinary use”
OIE veterinary use classification
WHO human use classification
8

## Slide 9
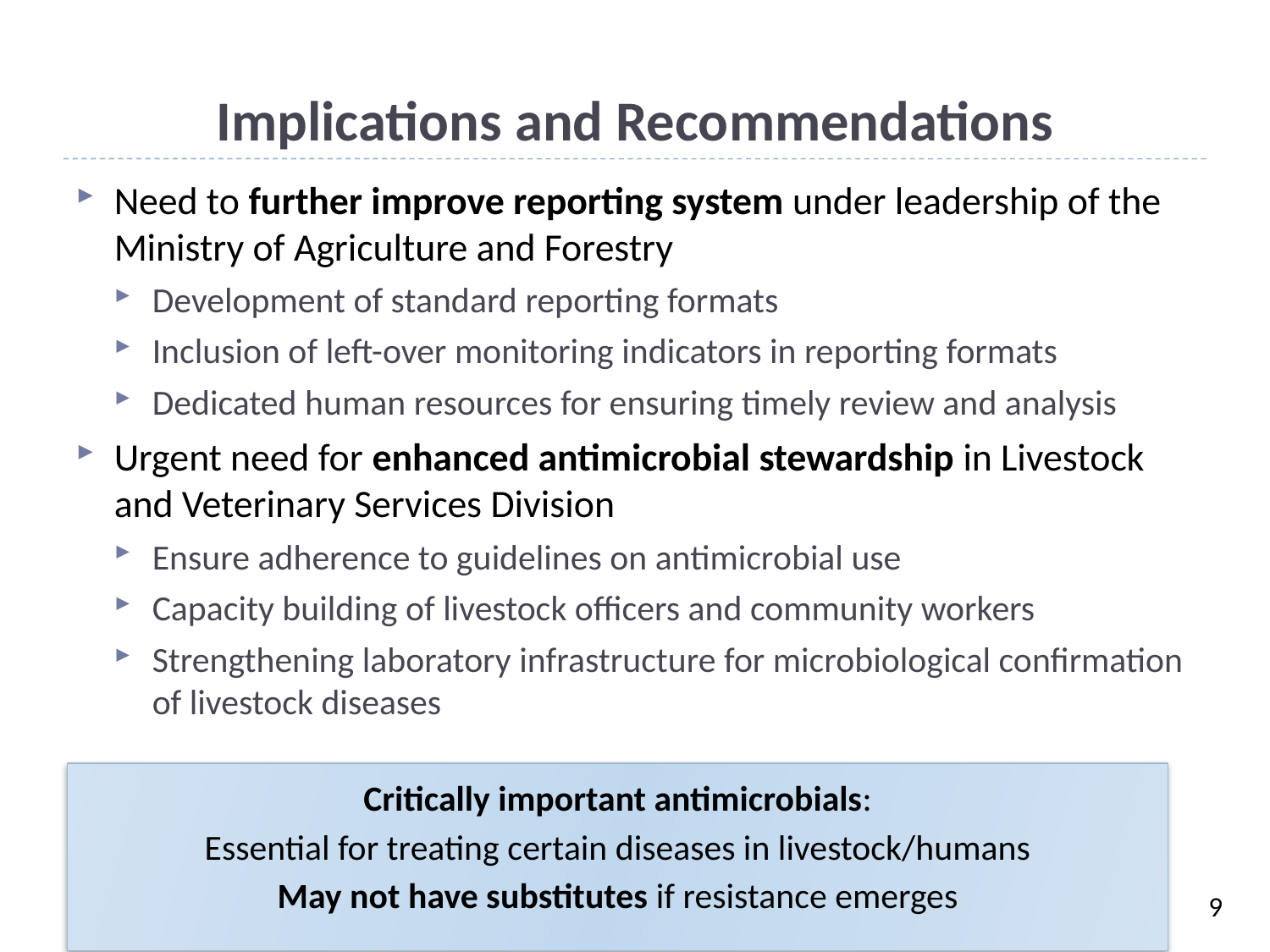

# Implications and Recommendations
Need to further improve reporting system under leadership of the Ministry of Agriculture and Forestry
Development of standard reporting formats
Inclusion of left-over monitoring indicators in reporting formats
Dedicated human resources for ensuring timely review and analysis
Urgent need for enhanced antimicrobial stewardship in Livestock and Veterinary Services Division
Ensure adherence to guidelines on antimicrobial use
Capacity building of livestock officers and community workers
Strengthening laboratory infrastructure for microbiological confirmation of livestock diseases
Critically important antimicrobials:
Essential for treating certain diseases in livestock/humans
May not have substitutes if resistance emerges
9

## Slide 10
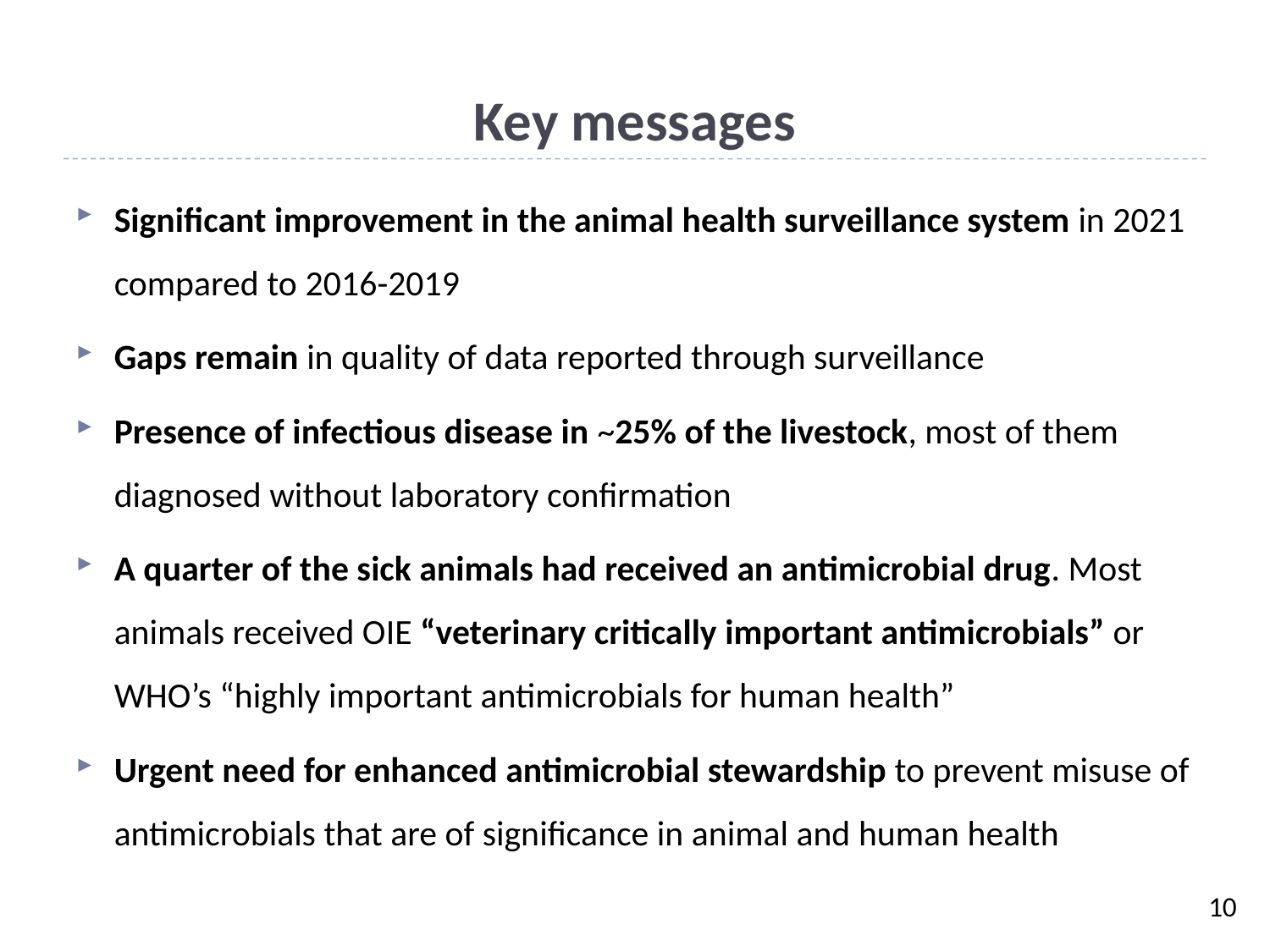

# Key messages
Significant improvement in the animal health surveillance system in 2021 compared to 2016-2019
Gaps remain in quality of data reported through surveillance
Presence of infectious disease in ~25% of the livestock, most of them diagnosed without laboratory confirmation
A quarter of the sick animals had received an antimicrobial drug. Most animals received OIE “veterinary critically important antimicrobials” or WHO’s “highly important antimicrobials for human health”
Urgent need for enhanced antimicrobial stewardship to prevent misuse of antimicrobials that are of significance in animal and human health
10

## Slide 11
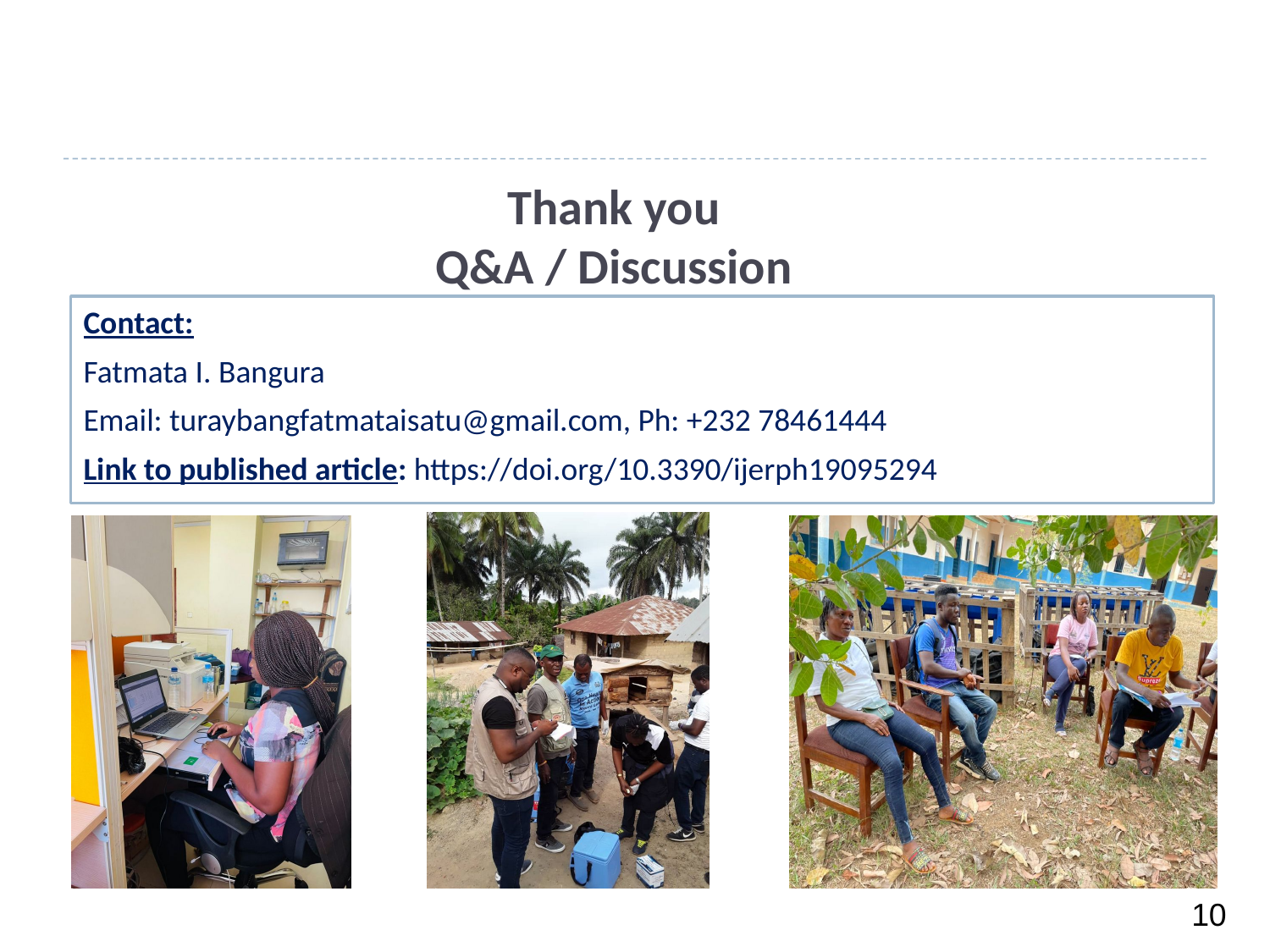

Thank you
Q&A / Discussion
Contact:
Fatmata I. Bangura
Email: turaybangfatmataisatu@gmail.com, Ph: +232 78461444
Link to published article: https://doi.org/10.3390/ijerph19095294
10
