# Supplementary material for: Improvement in the Surveillance System for Livestock Diseases and Antimicrobial Use Following Operational Research Studies in Sierra Leone January–March 2023
Source: Trop Med Infect Dis. 2023 Aug 10;8(8):408. doi: 10.3390/tropicalmed8080408 (PMC10459562; doi:10.3390/tropicalmed8080408)
Supplement: Supplementary file 1 [file tropicalmed-08-00408-s001.zip › S1_Community_based_surveilance_form.pdf]

Community Base Surveillance: Individual Reporting Form

Community member name: **Consultation Date:**

Client came to me for consultatio **Village:** **Own? Y N**

I observed the symptomatic event: **District:** **Chiefdom:**

CAHW ID:

Animal	Symptoms	Cases							Symptoms	Cases						
		1	2	3	4	5	6	7		1	2	3	4	5	6	7
	Nasal discharge								Convulsions							
	Eye discharge								Anorexia (very thin)							
	Oral lesions/ulcers								(High) Fever							
	Feet lesions/ulcers								Salivation							
	Udder lesions/ulcers								Diarrhoea							
	Lameness of one leg								Abnormal lumps							
	Swollen tongue/throat								Loss of appetite							
	Dead with leg not stiff								Rapid respiration							
	Bleeding from natural openings								Cough							
	Abortion after 4th month								Mortality (death)							
**Cow**	Change in milk colour								Reduced milk production							
	Nasal discharge								Milk stained with blood							
	Oral discharge								Dead with leg not stiff							
	Eye discharge								Convulsions							
**Small Ruminants - Sheep -**	Oral lesions/ulcers								Swollen joint							
**Goats**	Feet lesions/ulcers								(High) Fever							

	Udder lesions/ulcers								Salivation							
	Anorexia (very thin)								Diarrhoea							
	Lameness of one leg								Abnormal lumps							
	Swollen tongue/throat								Loss of appetite							
	Bleeding from natural openings								Cough							
	Abortion after 4th month								Mortality (death)							
	Change in milk colour								Reduced milk production							
	Weakness								Milk stained with blood							
	Loss of appetite								Oral discharge							
	Ruffled feathers								Twisted head and neck							
	Loss of balance/paralysis								Dead with leg not stiff							
	(Green) Diarrhoea								Convulsions							
	Bleeding from natural openings								Anorexia (very thin)							
**Birds -Chicken -Duck -Fowl**	Nasal discharge								Abnormal lumps							
	Behaviour disorder								Mortality (death)							
	Abundant salivation								Loss of appetite							
	Progressive paralysis								Dead with leg not stiff							
**Dog**	Bleeding from natural openings								Anorexia (very thin)							
	(High) Fever								Convulsions							
	Loss of appetite								Dead with leg not stiff							
	Swollen tongue/throat								Convulsions							
	Diarrhoea								Udder lesions/ulcers							
	Oral discharge								Swollen joints							
	Nasal discharge								Anorexia (very thin)							
	Oral lesions/ulcers								Abnormal lumps							
	Feet lesions/ulcers								Mortality (death)							
**Pig**	Bleeding from natural openings								Abortion after 4th month							
**Other Events**	wildlife								Red skin							

	Domestic															

Date Of Report Submission:

Name Of Animal Supervisor:

Form #:
